# Proximity to explosive synchronization determines network collapse and recovery trajectories in neural and economic crises

**DOI:** 10.1073/pnas.2505434122

**Published:** 2025-10-30

**Authors:** UnCheol Lee, Hyoungkyu Kim, Minkyung Kim, Gabjin Oh, Pangyu Joo, Ayoung Park, Dinesh Pal, Irene Tracey, Catherine E. Warnaby, Jamie Sleigh, George A. Mashour

**Affiliations:** ^a^Department of Anesthesiology, University of Michigan Medical School, Ann Arbor, MI 48109; ^b^Center for Consciousness Science, Michigan Psychedelic Center, Neuroscience Graduate Program, University of Michigan, Ann Arbor, MI 48109; ^c^Division of Business Administration, College of Business, Chosun University, Gwangju 61452, Republic of Korea; ^d^Wellcome Centre for Integrative Neuroimaging, Oxford Centre for Functional MRI of the Brain, Nuffield Department of Clinical Neurosciences, Nuffield Division of Anaesthetics, University of Oxford, Oxford OX3 9DU, United Kingdom; ^e^Department of Anesthesiology, Faculty of Medical and Health Sciences, University of Auckland, Auckland 1023, New Zealand

**Keywords:** explosive synchronization proximity, criticality, phase transition, network dynamics

## Abstract

Complex systems such as the conscious brain and financial markets often operate near criticality, a regime that supports flexible and efficient function. When perturbed, however, rapid deviations from, and prolonged recovery to, criticality can severely disrupt these systems. The nature of such transitions depends on a system’s intrinsic phase transition type. Most systems in nature undergo continuous (second-order) transitions, but some approach a discontinuous, first-order transition known as explosive synchronization (ES). Systems nearer to first-order transitions are more unstable, losing criticality more rapidly and recovering more slowly. However, no existing method can directly determine from empirical data how close a system is to a first-order regime, limiting our ability to predict criticality transition patterns. Here, we introduce a physics-based framework that estimates a network’s ES proximity at the critical point. Using modified Stuart–Landau oscillator networks, we show that distinct critical dynamics emerge depending on the proximity to ES and that this measure predicts the temporal patterns of collapse and recovery under perturbations. We validated the generality of our computational findings with empirical data on network collapse and recovery, using human electroencephalogram recordings during general anesthesia and global stock market indices from 39 countries during the 2008 economic crisis. We demonstrated that rapid collapses and prolonged recoveries in both brain and stock market networks can be systematically predicted for neuronal and economic crises. These results provide crucial insights for designing resilient networks capable of withstanding perturbations and recovering quickly.

## Significance

Why do some systems collapse abruptly and recover slowly, while others remain resilient? We show that the difference depends on how close a system’s second-order phase transition is to the first-order (explosive) limit, quantified as explosive synchronization (ES) proximity. Incorporating this measure into conventional criticality analysis provides a way to predict a network’s behavior during crises. Using this approach, we demonstrate that the loss and recovery of consciousness under anesthesia, as well as the collapse and recovery of stock markets during the 2008 economic crisis, can be systematically predicted as either rapid or slow. This framework offers a unified, physics-based tool for anticipating transition trajectories across complex systems.

Many physical and biological complex systems in a normal healthy state operate near criticality, which is a functionally optimal state at the edge of phase transitions that maximizes their capacity for information processing, integration, and sensitivity to environmental changes (1–5). When systems deviate from this critical state, they lose optimal functionality. In particular, abnormal transitions such as premature criticality loss and prolonged recovery can cause severe damage to systems. For example, patient complications can arise from excessive sensitivity to or prolonged recovery from anesthesia, a perturbation to the brain (6). Similarly, a rapid financial market crash or prolonged recovery can negatively affect a country’s economy (7). Predicting these adverse transitions before they occur is crucial, yet no established methods currently exist to anticipate the trajectory of criticality loss and recovery in such crises.

Previous work has concentrated on various ways of measuring how *far* a system has deviated from its zone of criticality. This distance is essential for understanding the extent to which a functional optimum is compromised under perturbations (8, 9). Furthermore, identifying whether this deviation pushes the system toward a subcritical (disordered, incoherent) or supercritical (ordered, synchronized) state is crucial for assessing the consequences of these external disturbances and for developing strategies to restore criticality (10, 11). Knowing both the *distance* from criticality and the *direction* of deviation informs us about how far and in what manner a system has drifted from its optimal state during crises, which defines the degree of criticality.

**Fig. 1.**
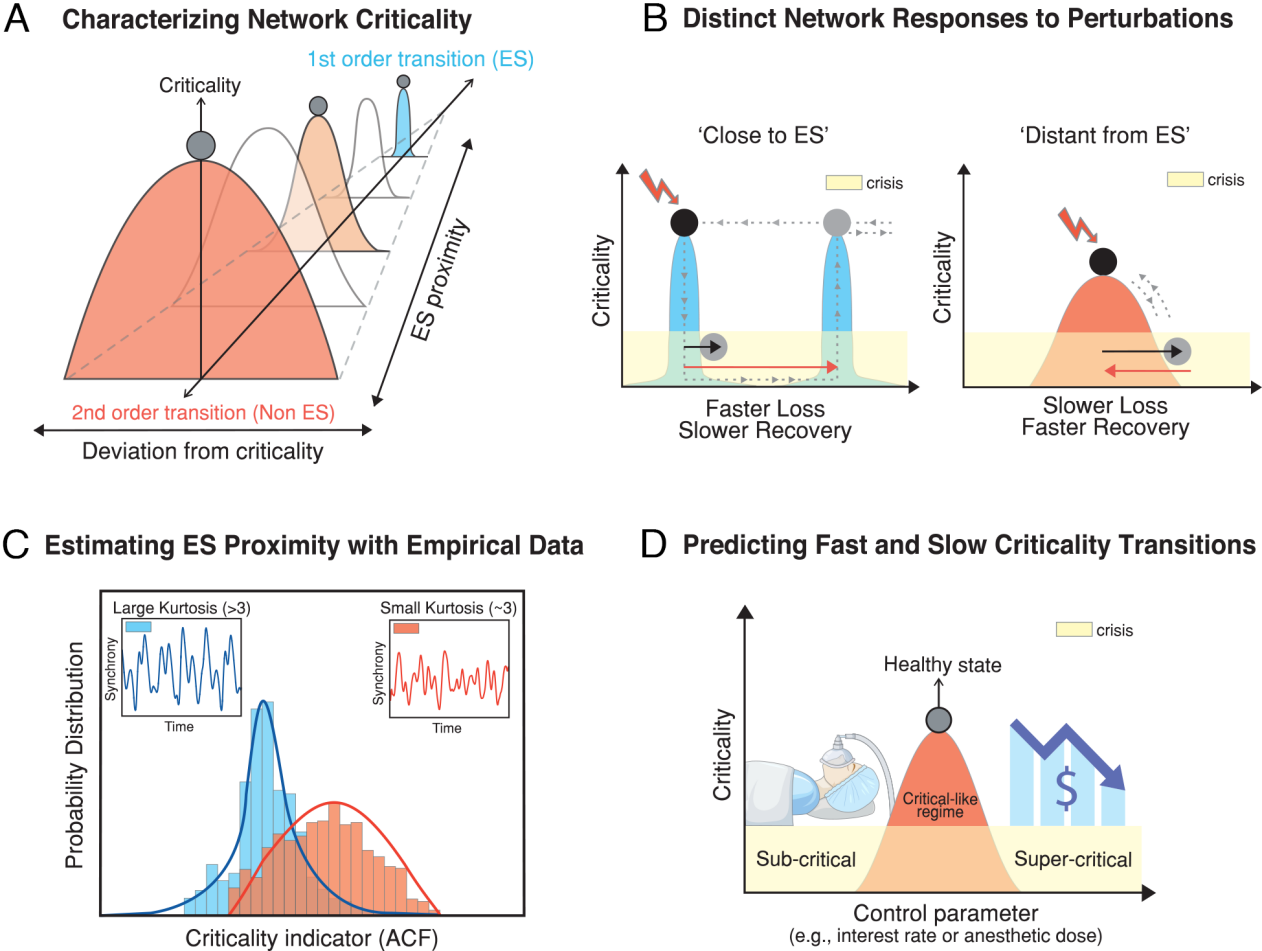
Network ES proximity, resilience, and recovery under perturbation. (*A*) Networks at healthy and normal states stay near critical points, while network crisis is characterized by a deviation from the critical point. Individual networks have diverse proximities to ES, i.e., first-order phase transition, resulting in larger interindividual variability in criticality loss and recovery. (*B*) Response behaviors to perturbations differ according to ES proximity: networks with higher ES proximity are more susceptible at critical points and show greater internal resistance, manifested as hysteresis, leading to faster criticality loss and slower recovery. Black and red arrows mark approximate times of criticality loss and recovery, respectively. (*C*) Networks with close or distant ES proximities exhibit distinct dynamics at critical points or critical-like regimes, which can be estimated through time series signals. (*D*) Real-world complex networks, such as the human brain and financial networks, operate near critical points in healthy and normal states. When these systems deviate from criticality, as during anesthetic induction or financial crises like 2008, precrisis ES proximity significantly determines the rates of consciousness or market collapse and recovery.

However, we should also be cognizant that a system’s response to perturbations, such as rapid loss of criticality and slow recovery, is shaped by the system’s inherent phase transition type, which often lies on a broader spectrum of second-order (gradual/continuous) transitions with a first-order (abrupt/discontinuous) limit (12). Although the general features of first- and second-order transitions are well established, the dynamic differences at the critical points of these second-order transitions, particularly as they approach the first-order limit, remain poorly understood. Fig. 1 illustrates these concepts in a two-dimensional state space, with one axis representing phase-transition types, ranging from second-order to first-order, and the other representing the distance from maximal criticality for each phase transition type. Since normal, healthy dynamical systems tend to stay near critical points, characterizing the distant dynamics at these points for systems with different phase transition types may allow us to predict response behavior during crises, including criticality loss, recovery trajectories, and adverse transitions (premature, prolonged) before they occur.

To test these expectations in complex networks, we extend our framework to network dynamics by examining Explosive Synchronization (ES), a first-order phase transition manifestation in networks that is characterized by an abrupt shift from an incoherent to a synchronized state. The mechanisms driving ES and the network conditions that induce it—such as heterogeneous frequency and degree distributions, higher-order connectivity, adaptive feedback, etc.—are well established (13–15). Given that these conditions are ubiquitous in real-world networks, ES is considered a universal phenomenon and has been widely studied as the underlying mechanism behind abrupt transitions in systems like power-grid failures, epileptic seizures, sudden wakefulness during light anesthesia, and hypersensitive brain activity in chronic pain (14, 16–19).

In our computational model, we introduced ES proximity as a graded measure that quantifies how closely a network’s second-order phase transition approaches the first-order limit (ES). ES proximity is assessed at the network’s critical point before transitions occur. We found that networks with higher ES proximity at their critical points follow trajectories that increasingly resemble first-order transitions, losing criticality more rapidly and recovering more slowly, as they approach ES. Specifically, we investigate the following questions: 1) Do networks with varying ES proximities exhibit distinct dynamics at their critical points, allowing us to infer ES proximity from the dynamics? 2) Can a network’s ES proximity, measured before a crisis, predict the speed and duration of network collapse and recovery during and after the crisis? 3) How do network structure and dynamics influence ES proximity and transition patterns under external perturbations? 4) Is ES proximity a better predictor of transition patterns during crises than conventional indicators based on deviation from criticality?

To validate our computational model empirically and assess its generality, we applied the approach to real-world complex networks, specifically brain and financial systems, to predict abnormal transitions after perturbation (whether premature or prolonged). This cross-domain approach successfully predicted consciousness loss and recovery patterns in brain networks (using 32-channel EEGs from 16 human subjects under anesthesia) as well as collapse and recovery patterns in stock market networks (comprising approximately 100 companies each for 39 countries) during the 2008 financial crisis.

## Results

### The Proximity of a Complex Network to ES Determines Rapid or Prolonged Transitions Near a Critical Point: A Computational Model Study.

We examined how a network’s proximity to ES affects dynamics near critical points and the response to perturbations. The conventional Stuart–Landau model, a simplified representation of an oscillator near a Hopf bifurcation, has been used to study network dynamics near and far from critical points (20). We employed a modified Stuart–Landau model with two new parameters: one varying ES proximity and the other varying perturbation strength (21). This model allows us to characterize network dynamics at critical points for different ES proximities and analyze their responses, quantifying the time for critical state loss and recovery. We further explored how different network topologies—random, scale-free, and small-world—affect these dynamics.

The modified Stuart–Landau model is defined as follows:

$$\begin{array}{c} \dot{z_{j}}\left(t\right)=\left\{\lambda_{j}+i\omega_{j}-{\left|z_{j}\left(t\right)\right|}^{2}\right\}z_{j}\left(t\right)\\ \;\;\;\;\;\;\;\;\;\;\;\;\;\;\;+R_{j}^{Z}S\sum_{k=1}^{N}\;A_{\mathrm{jk}}K_{\mathrm{jk}}z_{j}\left(t-\tau_{\mathrm{jk}}\right)+\beta \xi_{j}\left(t\right)+u\left(t\right), \end{array}$$

$$j=1,2,\cdots ,N\;\;\;u\left(t\right)=\left\{\begin{array}{c} p,t_{1}<t<t_{2}\\ 0,\mathrm{otherwise} \end{array}\right..$$

In the model, each node in the network is represented as a complex variable $z_{j}=r_{j}e^{i\theta_{j}}$, where $r_{j}$ and $\theta_{j}$ are the amplitude and phase of oscillator *j*. The dynamics are governed by each node’s natural frequency $\omega_{j}$, amplitude decay $\lambda_{j}$, structural connectivity $A_{\mathrm{jk}}$, time delays $\tau_{\mathrm{jk}}$, and coupling strength *S.* Gaussian white noise $\xi_{j}\left(t\right)$ with variance β = 0.05 is added to capture stochastic fluctuations. The connection matrix A depends on the chosen network topology, with $A_{\mathrm{jk}}=1$ if nodes are connected and 0 otherwise. The adaptive feedback term $R_{j}^{Z}$ represents recursive interactions among nodes. To study the network’s state transition behavior, we applied global pulsatile stimuli *u(t)* of varying intensity *p* and durations *T* = *t*_2_ − *t*_1_, delivered at random times. Each simulation tested multiple frequency configurations and stimulus trials to assess criticality collapse and recovery behavior.

In our model, the strength of the feedback process serves as the primary control parameter for modulating a network’s phase transition type from non-ES (gradual, second-order) to ES (abrupt, first-order). Filatrella et al. first investigated how a feedback term can induce this shift, using a generalized Kuramoto model focused solely on phase dynamics (22). In our previous work (21), we extended this approach by introducing a feedback-modulated Stuart–Landau model that incorporates both amplitude and phase dynamics, enabling us to describe the amplitude dynamics of EEG and stock volatility. Through a mean-field analysis, we demonstrated that increasing feedback strength introduces internal resistance to state changes, resulting in hysteresis and an ES transition. This mechanism was further generalized by Kuehn et al., who mathematically showed that incorporating additional control parameters, such as feedback, structural heterogeneity, or high-dimensional connectivity, etc., can generate internal resistance to synchronization transitions and shift a network’s phase transition type to ES. This shift is considered universal and typically characterized by subcritical pitchfork or saddle-node bifurcations (15).

Unlike non-ES networks without such resistance, ES networks involve competition between two opposing forces: one promoting change in synchronization and the other resisting change. At critical points, these two forces are balanced, resulting in networks that are highly sensitive to perturbations and prone to abrupt transitions. This is the core mechanism of ES. Based on these dynamics, we hypothesize that networks with higher ES proximity are more susceptible to perturbations and therefore lose their critical states more easily. However, due to their internal resistance and bistability, these networks require a longer recovery period after the perturbation is stopped.

To test this hypothesis, we constructed models comprising 78 interconnected Stuart–Landau oscillators representing a real-world complex network (a diffusion tensor image-informed human brain network), and 1,000 interconnected Stuart–Landau oscillators for small-world, random, or scale-free networks with 4,000 structural links. Each oscillator simulates node activity and interactions with linked nodes. We ran 100 simulations for each of the seven ES proximities, varying initial conditions. To identify each network’s critical point, we tested the pair correlation function (PCF) and autocorrelation function (ACF) of the instantaneous order parameters. The PCF measures the variance of collective phase synchronization fluctuations, while the ACF measures the temporal memory of these fluctuations. Both the PCF and ACF reach maximal values at critical points (23, 24). In this simulation study, we utilized the peak PCF to identify critical points because it is nonparametric and more effective at detecting peaks than the ACF in our model. We validated the criticality of the peak PCF with widely used criticality indicators, including finite-size scaling, detrended fluctuation analysis, deviation from criticality coefficient, branching ratio, and rescaling analysis (*SI Appendix*, Figs. S5–S8). In particular, the finite-size scaling analysis shows that the modified Stuart–Landau model at the peak PCF belongs to the 2D percolation universality class (*SI Appendix*, Table S9).

**Fig. 2.**
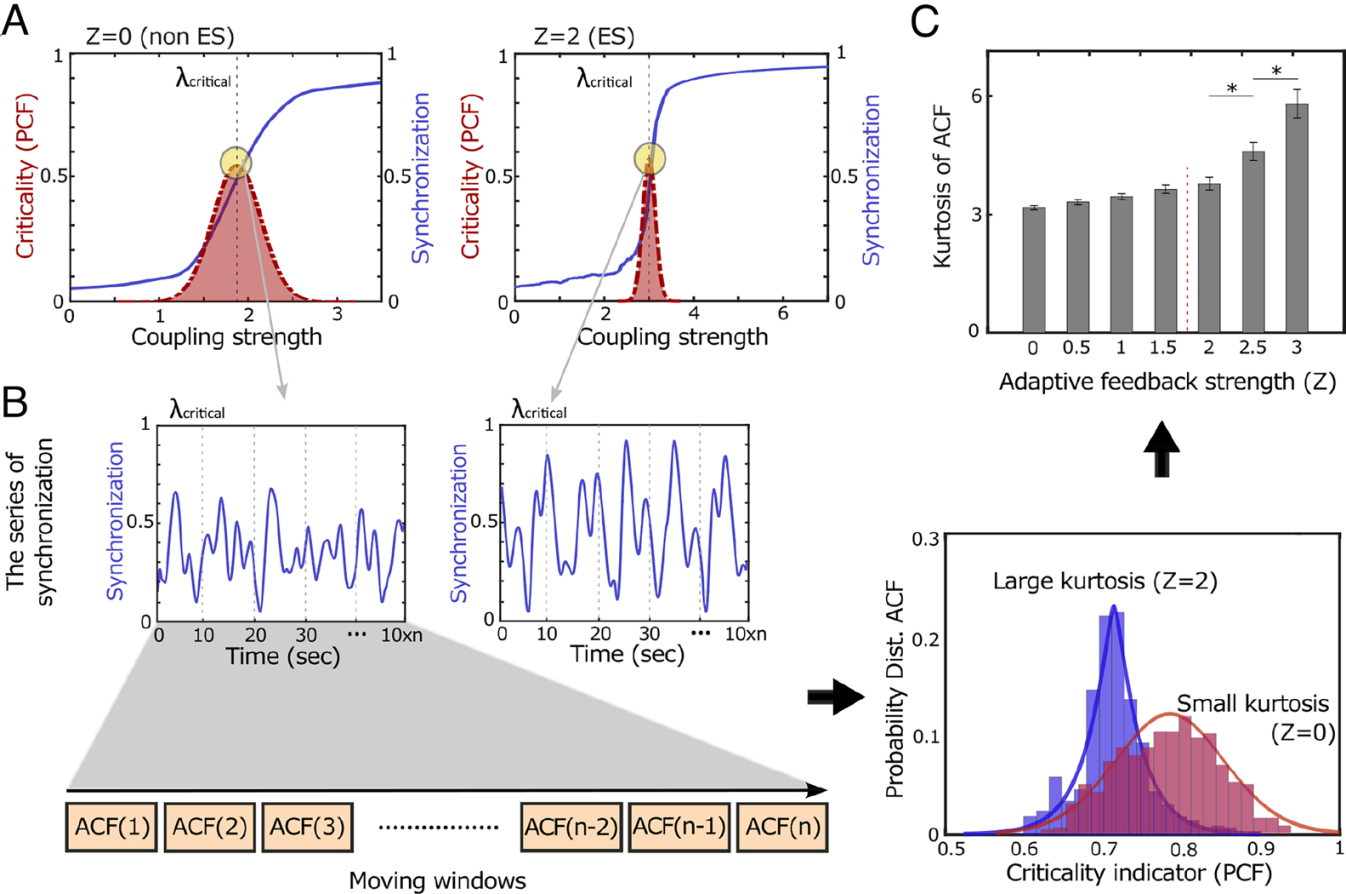
Distinct network dynamics at critical points with varying ES proximity. (*A*) Networks with close (Z = 2) and distant (Z = 0) ES proximities exhibit distinctive network dynamics near their critical points. The network with higher ES proximity (Z = 2) undergoes a steeper phase transition near its critical point than the network with lower ES proximity (Z = 0). Critical points were identified based on the maximal PCF of instantaneous order parameters (indicated by circles). The red dashed line indicates the PCF values at each coupling strength. (*B*) A moving window technique was employed to analyze the variability of the ACF of order parameters, distinguishing the close and distant ES proximities. (*C*) The ACF distribution of a close ES proximity (Z = 2) has a larger kurtosis compared to that of the lower ES proximity network (Z = 0). The distinct ACF distributions, which reflect different network dynamics at critical points, demonstrate the potential to estimate the ES proximity using time series data of a complex dynamical network.

In Fig. 2*A*, we illustrate the peak PCFs with yellow circles for Z = 0 and Z = 2. The network with higher ES proximity (Z = 2) exhibits a steeper synchronization transition at the critical point than the one with lower ES proximity (Z = 0). We analyzed time series signals across various ES proximities and found that networks with closer ES proximities displayed more variability in sequential ACF values (Fig. 2*B*), reflecting intermittent and bistable transitions near their critical points (*SI Appendix,* Fig. S1 *A*–*D*). This indicates that these networks tend to produce uncommon ACF values, including extremely high and low ones, leading to fat tails in ACF distributions (Fig. 2*C*). The kurtosis of the ACF distribution, which reflects these fat tails, is correlated with the adaptive feedback strength Z. As Z increases and begins to influence network dynamics near the critical point (Z > 2), the kurtosis of the ACF distribution increases significantly (*P* < 0.05; *SI Appendix*, Tables S1 and S2 for the statistical tests). From Z > 2, the network dynamics exhibit heavy tails (kurtosis greater than 3). This suggests that the kurtosis of the ACF distribution at a critical point could serve as an indicator of ES proximity. We also obtained similar results with the kurtosis of the PCF (*SI Appendix,* Fig. S1 *E* and *F*). However, since the kurtosis of the ACF aligns more consistently with the real-world data we tested, our presentation was focused on the ACF results.

**Fig. 3.**
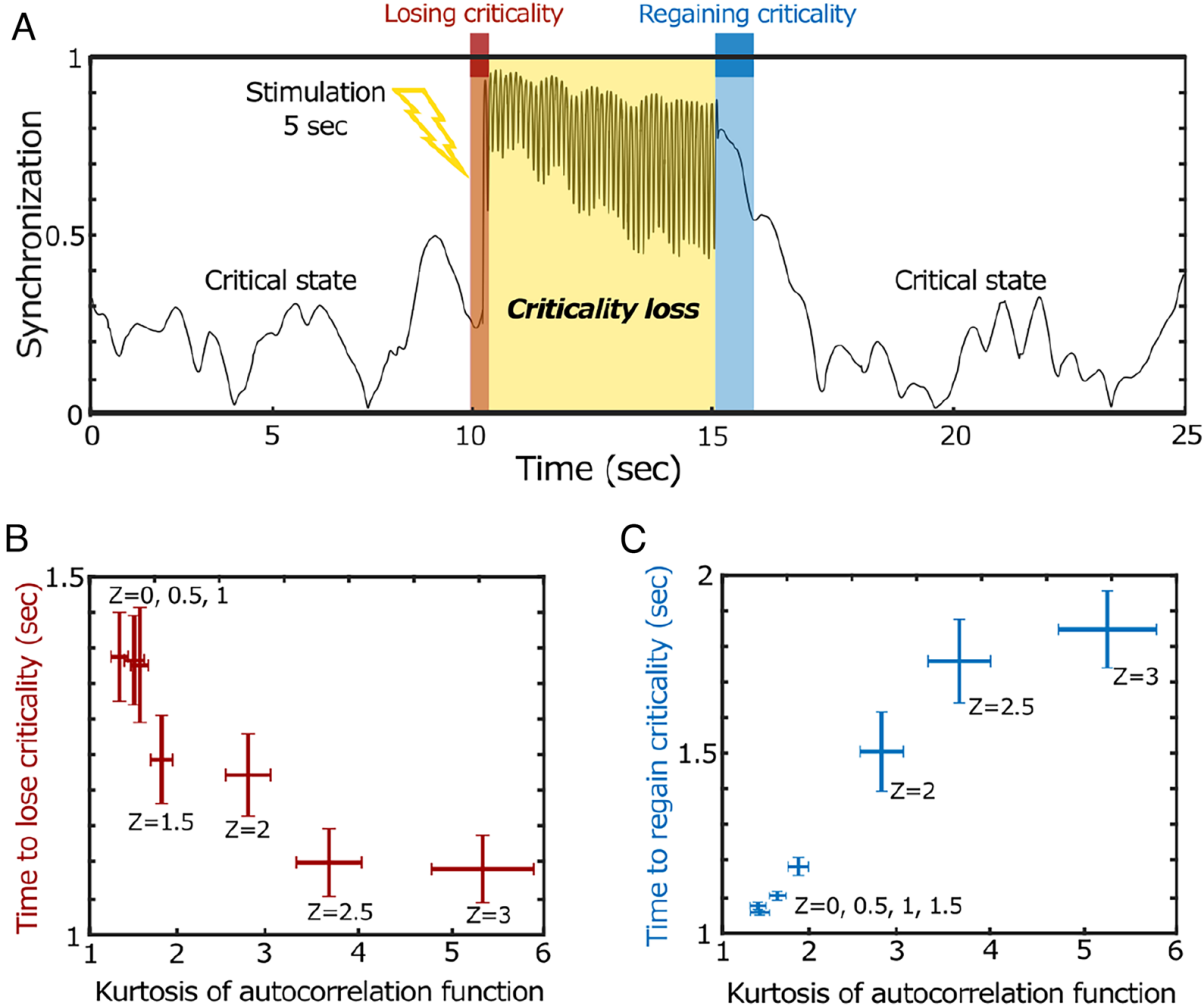
The influence of ES proximity on the critical state loss and recovery under external perturbation. (*A*) A network with higher ES proximity (Z = 2) presents characteristic critical state loss and recovery patterns. Under external perturbation, the network loses and regains its baseline critical state. The times to lose (red zone) and regain (blue zone) the baseline states were determined by the times when the network’s ACF values cross-over and return to within three SD of the ACF values in the baseline state. (*B*) The kurtoses of baseline ACF distributions are negatively correlated with the times to baseline critical state loss, indicating that networks with higher ES proximity (a larger Z) are more prone to faster critical state loss. (*C*) Conversely, the kurtoses of baseline ACF distribution are positively correlated with the recovery time. A network with higher ES proximity (a larger Z) exhibits slower critical state recovery. Error bars indicate SE of 100 simulations.

To test how networks with different ES proximities respond over time, we introduced external perturbations u(t) globally. Fig. 3*A* illustrates how these perturbations disrupt baseline dynamics at a critical point and how the network returns to its baseline state as the perturbation fades. In our model, the fixed coupling strength at the critical point ensures the network self-organizes and returns to its baseline state after the perturbation ends. We measured the time for the network to deviate from and return to the baseline state by defining a zone as three times the SD of ACF values at baseline. We then calculated correlations between the kurtosis of baseline ACF values before perturbation and the time required for the network to lose and regain its baseline dynamics.

Fig. 3*A* shows the response of a network with higher ES proximity (Z = 2) to an external perturbation. Following a 5-s perturbation at u(t) = 10, the network exhibits an abrupt deviation from the baseline state, followed by gradual recovery. Fig. 3 *B* and *C* demonstrate significant correlations between the kurtosis of ACF values and the times taken for critical state loss and recovery. We observed that the kurtosis of baseline ACF is negatively correlated with the time of critical state loss and positively correlated with recovery time. The robustness of the results was confirmed by testing various strengths u(t) (20, 40, 60, 80, and 100, *SI Appendix,* Fig. S2) and network structures (random, scale-free, small-world, *SI Appendix,* Fig. S3) (*SI Appendix,* Fig. S3). Additionally, we tested finite-size effects using random networks of varying node numbers (25, 50, 75, 100, 150, 250, and 500 nodes) and confirmed the robustness of the results (*SI Appendix,* Fig. S4). Overall, the results suggest that networks with higher ES proximity lose their baseline critical state more quickly but take longer to recover. We further show that only ES proximity predicts the fast or slow criticality loss and recovery under perturbation, whereas conventional indicators based on deviation from criticality (e.g., deviation from criticality coefficient and Hurst exponent) do not (*SI Appendix,* Fig. S13).

### Proximity to ES of Brain Networks during Baseline Consciousness Determines the Temporal Course of Anesthetic-Induced State Transitions.

General anesthesia represents a neural “crisis” where functional brain networks are profoundly perturbed, and the brain deviates from a critical state (25–27). We hypothesized that early or delayed transitions in loss and recovery of consciousness might be governed not only by the pharmacokinetics of anesthetics but also by the type of phase transition in functional brain networks. According to our model, we expected that the ACF kurtosis of the electroencephalogram (EEG) data recorded in a resting state, which is a surrogate for the ES proximity of the brain network, may predict the temporal course of state transitions at the boundaries of consciousness. Due to the high susceptibility and hysteresis of an ES network, we also expected that brain networks with a large kurtosis of ACF may exhibit a fast loss and slow recovery of consciousness during anesthetic transitions.

**Fig 4.**
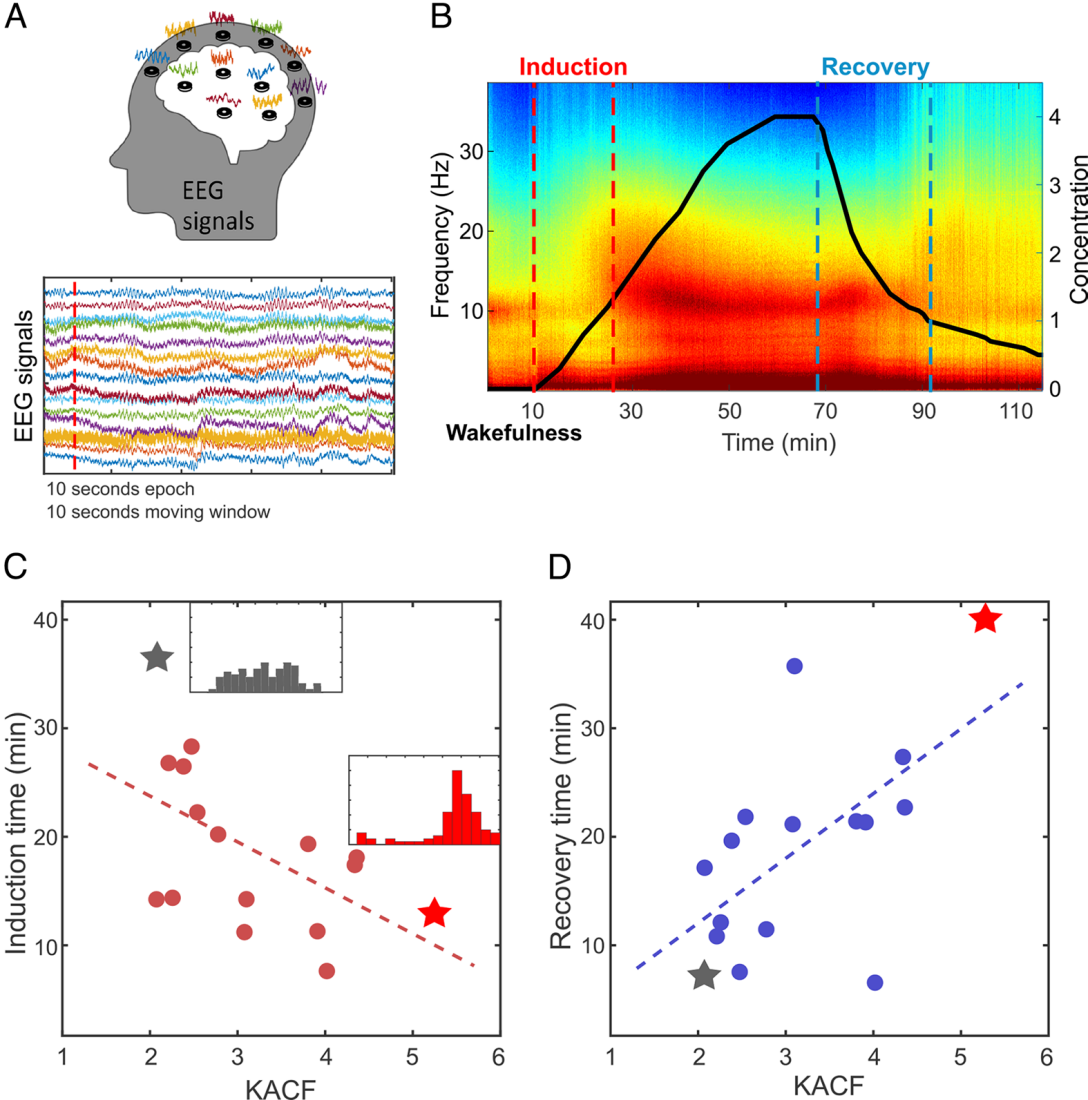
The ES proximity of human EEG in a conscious resting state significantly correlates with the induction time and the recovery time in general anesthesia. (*A*) Thirty-two-channel EEG signals during baseline consciousness, induction of anesthesia, unconsciousness, and recovery states were analyzed to test the relationship between ES proximity in conscious brains and fast/slow state transitions induced by the general anesthetic propofol. (*B*) The spectrogram shows a significant change in the spectral content of the EEG along with state transitions in anesthesia. The anesthetic induction and recovery times were defined by the time intervals between the injection of propofol and the loss of responsiveness (red dotted lines) to a verbal command (induction) and between the end of injection and the recovery of response (blue dotted lines) to a verbal command (recovery). The solid line indicates the modeled effect-site concentration of propofol in the volunteer’s brain. The kurtosis of ACF calculated with the baseline EEG shows a significant negative correlation with the induction times (*C*) for 16 subjects. Conversely, the kurtosis of the ACF of the baseline EEG positively correlates with the recovery time (*D*).

To test the hypothesis, we analyzed data from 16 healthy human subjects who had 32-channel EEG recorded during anesthetic state transitions. Fig. 4*A* illustrates the EEG electrodes and signals. Fig. 4*B* presents a single subject’s EEG spectrogram, and the states studied: resting state with eyes closed (10 min), induction period (start of anesthetic delivery to loss of consciousness), unconscious period (loss of consciousness to maximal anesthetic concentration), and recovery period (maximal anesthetic concentration to the recovery of consciousness). Times to loss and recovery of consciousness were determined by behavioral response to verbal command. Despite maintaining the same anesthetic effect-site concentration across subjects, the times of consciousness loss and recovery were variable (see y-axes in Fig. 4 *C* and *D*).

We calculated the kurtoses of ACFs using 3-min, resting-state EEG after preprocessing and noise treatments (See *SI Appendix*, Section S2 for details). First, we applied band-pass filtering to isolate the alpha-frequency band (8 to 13 Hz) and extracted the instantaneous phases with the Hilbert transform. Using these phases, we calculated the instantaneous order parameters for the EEG signals. To analyze the kurtosis of ACF, we employed a moving window on the sequence of instantaneous order parameters. Specifically, ACFs with a time lag of 50 (reflecting the alpha dynamics at ~10 Hz and a sampling frequency of 500 Hz) were computed within 10-s windows with a 5-s overlap. Finally, we calculated the kurtosis of the ACFs from all windows. We then assessed the correlation between the kurtosis of ACFs in resting states and times of consciousness loss and recovery. The results showed significant negative and positive correlations between resting-state kurtosis of ACFs and times to consciousness loss (Spearman correlation coefficient: ρ = −0.67, *P* < 0.01) and recovery (Spearman correlation coefficient: ρ = 0.59, *P* < 0.01), respectively. These findings indicate that an individual brain’s ES proximity in baseline states, measured by kurtosis of ACFs in EEG networks, predicts the pattern of conscious state transitions at the boundary between consciousness and unconsciousness.

In additional analyses, we demonstrated that the conscious brain exhibits typical features of criticality, including long-range correlations (Hurst exponent > 0.5) and power-law scaling (deviation from criticality coefficient of ~0) (*SI Appendix,* Figs. S9 and S10). However, the criticality indicators did not account for the gradual and abrupt transitions in consciousness (*SI Appendix,* Figs. S13 and S14). This suggests that the criticality indicator can measure the deviation from brain criticality, but is not a good predictor of the trajectory of loss and recovery of consciousness during anesthetic state transitions.

### Precrisis Proximity to ES of Stock Market Networks Determines the Time Courses of Stock Market Collapse and Recovery Associated with the 2008 Economic Crisis.

**Fig. 5.**
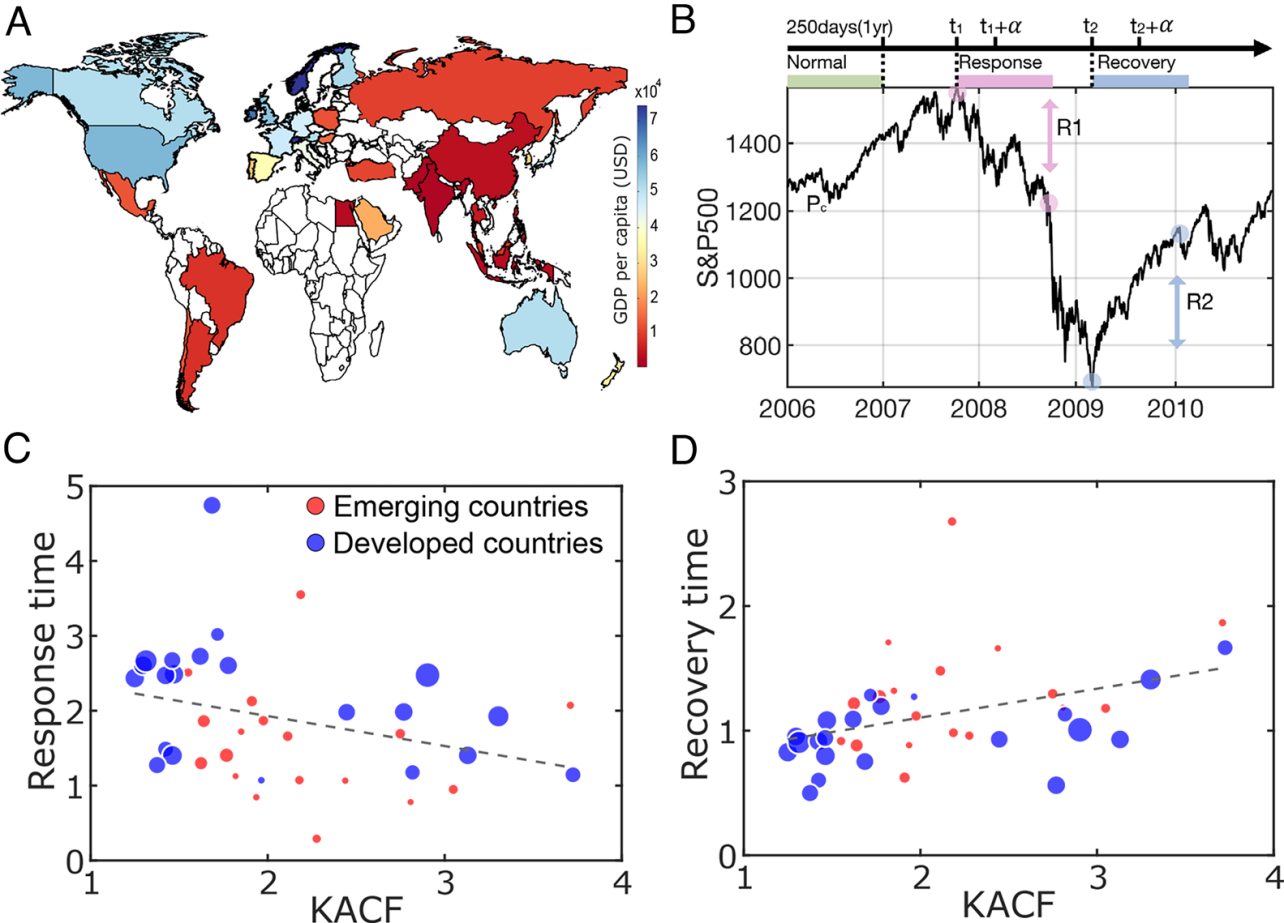
The ES proximities of stock market networks significantly correlate with the rates of market collapse and recovery during the 2008 economic crisis. (*A*) The thirty-nine countries analyzed in this study are mapped according to their 2006 GDP per capita (USD), with colors ranging from red to blue. (*B*) The market price of the S&P 500 index underwent a dramatic price change in the 2008-2009 economic crisis, which created natural epochs of baseline (precrisis), response (intracrisis), and recovery (postcrisis) periods. The ES proximity of a stock market network was calculated in the baseline period, and the response and recovery times were calculated with the market collapse (R1) and recovery (R2) rates. (*C* and *D*) The kurtosis of ACF in the baseline period is negatively correlated with the response time (*ρ* = −0.40, *P* < 0.001), and positively correlated with the recovery time (*ρ* = 0.49, *P* < 0.001). Blue and red circles represent developed and emerging countries, respectively, and the marker sizes are scaled by the country’s GDP.

To determine whether ES proximity is a general principle of state transitions during crises across diverse computational and real-world systems, we examined data from the 2008 economic crisis. This crisis was triggered by the collapse of the US subprime mortgage market, subsequently causing widespread banking system failure and, eventually, worldwide recession. Using the SandP Compustat Global database, we collected daily stock prices from 39 global equity markets from 2006 to 2010. Gross domestic product (GDP) per capita (in USD) is shown on the world map (Fig. 5*A*). Details of data collection and processing are found in *SI Appendix*, Table S3.

We defined analysis periods for each market: baseline, response, and recovery. Based on studies of the 2007-2009 Subprime Mortgage Crisis (28), we designated 2006 as the baseline period, preceding the crisis. We tested the robustness of our results by varying the baseline period (See *SI Appendix*, Table S4 for test results). We defined response and recovery periods for each country by calculating the market collapse rate (R1) and recovery rate (R2) during the 2008 crisis, which was triggered by the collapse of Lehman Brothers in September 2007. Recession periods were identified using recession indicators from the Organization of Economic Cooperation and Development. We first identified the maximum stock price during the recession period and evaluated the collapse rate over a period α, reflecting the price drop from the maximum. To assess the recovery rate, we identified the minimum price during the crisis and calculated how much the stock price rose from that minimum over the same period α (Fig. 5*B*). Considering the large difference in the price ranges among countries, we normalized market collapse and recovery rates, R1 and R2, for each country (*c*) as follows.

$$R1\left(c\right)=\frac{P_{c}^{m}\left(t_{1}\right)-P_{c}^{m}(t_{1}+\alpha )}{P_{c}^{m}\left(t_{1}\right)-P_{c}^{m}\left(t_{2}\right)},\;R2\left(c\right)=\frac{P_{c}^{m}\left(t_{2}+\alpha \right)-P_{c}^{m}\left(t_{2}\right)}{P_{c}^{m}\left(t_{1}\right)-P_{c}^{m}\left(t_{2}\right)},$$

where $P_{c}^{m}\left(t\right)$ is the stock market index of country *c* and time $t=1,2,3,\ldots T.\;t_{1}=\{t\in T:P_{c}^{m}(t)\}$, $t_{2}=\{t\in T:P_{c}^{m}(t)\}$, indicate dates of maximal and minimal stock prices, respectively. Because the 39 stock markets have diverse dynamics, we tested several time periods, α={40, 60, 80, 100, and 120 days}, to calculate collapse and recovery rates. 40 and 120 d correspond approximately to 2 and 6 mo, considering market closures. Here, we chose α of 100 d, an appropriate period to reflect the scale of price changes during the crisis. However, the results were not sensitive to α (See *SI Appendix*, Table S5 for test results). Finally, we applied logarithms to the inverse of the market collapse and recovery rates for direct comparison with the EEG study’s induction and recovery time.

$$\mathrm{Response}\;\mathrm{time}\left(c\right)=ln(\frac{1}{R1\left(c\right)}),\;\mathrm{Recovery}\;\mathrm{time}\left(c\right)=ln(\frac{1}{R2\left(c\right)}),$$

With this transformation, a shorter response/recovery time (i.e., a faster response/recovery rate) corresponds to a shorter induction/recovery time in the EEG study. The financial network analysis showed that the kurtosis of ACFs in the baseline period negatively correlates with the market response times (Spearman coefficient, ρ = −0.40, *P* < 0.001, Fig. 5*C*); conversely, the kurtosis of ACFs positively correlates with market recovery times (Spearman coefficient, ρ = 0.49, *P* < 0.001, Fig. 5*D*). In other words, stock markets with higher ES proximity at baseline showed faster market collapse and slower recovery after the crisis. This is consistent with our computational predictions and empirical study of anesthetic state transitions. Moreover, we found the kurtosis of ACF (lag2) significantly correlates with the GDP per capita (ρ = −0.36, *P* < 0.05) (See *SI Appendix*, Table S6 for the test results). These results indicate that the state transitions in emerging markets–for instance, Thailand, Philippines, and Indonesia, which are classified as Morgan Stanley Capital International emerging markets–are relatively closer to ES than those in developed markets and, therefore, are highly unstable during economic crises.

Additionally, the daily stock price data in the precrisis period exhibited finite-size scaling (*SI Appendix,* Fig. S11) with distinct universality classes across markets (US: Mean-field, Canada: 3D percolation, Korea: intermediate between Mean-field and 3D percolation), as well as a long-range temporal correlation (Hurst exponent > 0.5) and other hallmarks of criticality (*SI Appendix,* Fig. S12). However, the long-range temporal correlation did not account for the fast and slow market collapse and recovery (*SI Appendix,* Fig. S15). This highlights the unique role of ES proximity in predicting market response to economic crisis. Notably, the power-law scaling analysis of avalanche size and duration was not applied due to insufficient daily stock price data for calculating avalanche statistics.

## Discussion

We investigated how a network’s proximity to ES influences the time courses of network collapse and recovery during computational, neural, and financial crises. In our computational model, networks with different ES proximities exhibited distinct dynamics at their critical points, reflected in the kurtoses of ACF values. We found that the kurtosis of ACF values before perturbation was negatively correlated with the time to critical state loss and positively correlated with the time to recovery. This indicates that proximity to ES, a first-order transition limit within a broader spectrum of second-order transitions, shapes the speed of network collapse and recovery. Specifically, networks closer to ES tend to lose their critical state quickly and recover more slowly. This relationship was robust across diverse model networks (scale-free, small-world, and random) and real-world networks (human brain networks during anesthesia and stock market networks during the 2008 global economic crisis). By contrast, conventional criticality indicators, such as deviation from criticality coefficient and Hurst exponent, did not account for fast or slow state transitions in the computational, anesthesia, or economic data.

### ES Proximities of Human Brain and Stock Market Networks.

Although the brain and financial networks are composed of distinct elements and operate on different principles, both share features as nonequilibrium systems where flows of energy, matter, and information are continuously introduced. The brain is a hierarchical network of neurons that process signals for sensory perception, motor control, cognition, and emotions. Financial networks, on the other hand, interconnect banks, investors, and institutions through assets and investments to maintain market stability and efficiency. Despite their differences, both networks reside near their critical states under normal conditions (1–3, 29–31). We hypothesized that each network’s response to significant perturbations depends on its inherent proximity to a first-order phase transition (i.e., proximity to ES).

Previously, we found evidence of ES in the brain networks of individuals under light anesthesia (17). Anesthesia reconfigures functional brain networks, decreasing global efficiency and enhancing modularity, which suppresses network synchronization and prompts ES conditions in some subjects (17, 32). Our EEG analysis showed that ES proximity in a resting state is significantly correlated with both induction and recovery times associated with propofol anesthesia, especially in the alpha band (8 to 13 Hz). Alpha waves coordinate hierarchical neural activities, facilitating cognitive processing and top–down control (33, 34), filtering out irrelevant sensory inputs, enhancing attentional focus, and transferring information globally via traveling waves (35, 36).

Our EEG analysis showed that only the alpha network’s ES proximity in the resting state reliably predicts both induction and recovery times. This finding suggests that the network mechanism driving diverse brain state transitions at the edge of consciousness prominently operates within globally networked alpha waves. Given that ES proximity significantly influences the brain’s response to perturbations, we propose that individual variations in a brain network’s proximity to ES should be considered an important factor for effectively predicting and modulating brain states through various interventions (pharmacological, electrical, magnetic, etc.).

We applied the same method to financial networks during an economic crisis to test its generality. Significant correlations were found between the kurtosis of ACF values in the precrisis period, the market response time during the crisis, and the recovery time afterward. These results aligned with both our computational model predictions and observations related to anesthetic state transitions, suggesting that ES proximity, as measured by daily stock prices, could serve as a market index for characterizing financial markets and potentially forecasting market collapse and recovery. Furthermore, ES proximity in the precrisis period was negatively correlated with a country’s GDP per capita, indicating that markets with higher ES proximity tend to have lower GDP. This implies that emerging markets with lower GDP per capita are closer to a first-order phase transition and are more vulnerable to economic crises than mature markets. Our findings underscore the importance of further research into the relationship between stock market network dynamics, GDP, and market collapse and recovery during crises. These results provide insights into understanding economic crises through the lens of financial network dynamics and structure. Furthermore, it would be worth testing whether these findings generalize to other types of markets—such as bonds, foreign exchange, derivatives, and cryptocurrency—and to different sources of economic crises, such as the COVID-19 pandemic.

### How Feedback Processes Shift the Phase Transition Type.

In our previous study, we used mean-field analysis to show how feedback in the Stuart–Landau model introduces internal resistance to synchronization transitions (21). In the model, a local node’s effective coupling is defined as $k_{j}\cdot S\cdot R^{z}$, where *k_j_* is the degree of node *j* and *S* is the coupling strength. *R* is the global synchronization level feeding back into local dynamics. This feedback establishes a top–down influence, where the global network state shapes local synchronization. As a result, network state transitions become path-dependent: In low-synchrony states (*R* << 1), weak global feedback R suppresses local coupling, requiring a higher S to initiate synchronization. In contrast, in high-synchrony states (*R* ~ 1), strong feedback maintains local synchrony, making desynchronization more challenging. This asymmetry creates path dependence, leading to hysteresis: The system follows different trajectories for synchronization and desynchronization. As feedback strength Z increases, it enhances the top–down feedback influence; therefore, the hysteresis becomes wider, and the phase transition shifts from non-ES (second-order, gradual) to ES (first-order, abrupt). This mechanism also explains how feedback alters the stability of criticality near transition points, shaping the trajectory of criticality loss and recovery. Mathematically, this shift is explained by subcritical pitchfork or saddle-node bifurcations, where feedback induces bistable states and abrupt criticality transitions, unlike the smooth transitions seen in non-ES networks (15).

The analogy of theater clapping may help intuitively illustrate this point. Initially, when the audience is out of sync, each person claps independently, with minimal influence from others, reflecting weak top–down effects. As clapping becomes more coordinated, individuals begin to follow the group rhythm, and synchrony reinforces itself through this top–down influence. Once full synchronization is achieved, stopping the clapping requires a stronger external signal (e.g., the house lights turning on or the curtain closing), illustrating how the top–down effect creates internal resistance or inertia to state change and results in an abrupt transition. This is distinct from a gradual transition in clapping alone, which represents a second-order transition.

### Insights into Rapid and Prolonged System Collapse and Recovery Around the Time of Crisis.

First, research on catastrophic phase transitions, commonly known as “fold bifurcations” or “subcritical bifurcations” in bifurcation theory, has made significant theoretical progress (37–40). Empirical indicators like critical slowing down, increasing variance and correlation in space and time, and spectral reddening (shifting the highest frequency to a lower one) have demonstrated the potential to anticipate impending phase transitions in diverse systems, including socioecological, neurological, financial, and climate systems. Recent theoretical studies have highlighted the need not only to predict upcoming critical points but also to identify the type of bifurcation that governs the transition pattern near these critical points. For instance, a smooth transition (transcritical bifurcation), the emergence of oscillations (Hopf bifurcation), or an abrupt change to another attractor (fold bifurcation) could characterize the transition pattern. However, given the generality of the critical slowing-down phenomenon, these indicators are unable to distinguish the bifurcation types, limiting the capacity to estimate the transition patterns near critical points (41). In complex dynamical networks, the kurtosis of ACF offers a promising approach to address this limitation by estimating the proximity of a network’s phase transition type to a first-order transition, i.e., ES. We propose that combining the kurtosis of ACF with critical slowing-down indicators may enable the prediction of both impending critical points (abrupt transitions, often associated with system crises) and their associated phase transition types.

Second, previous theoretical models have identified network configurations that can induce ES (14). However, practical application is limited by the difficulty of obtaining precise network structures in real-world scenarios. The kurtosis of ACF, a signal-based ES proximity indicator, can estimate ES proximity using time series signals, making it valuable for monitoring changes in a network’s phase transition type over time. For instance, we applied this approach to sickle cell disease and found that ES proximity in brain networks progressively increases until a pain crisis occurs and then diminishes afterward, repeating unpredictably over weekly or monthly intervals (19). The kurtosis of ACF could be used to monitor changes in ES proximity and assess network vulnerability over time.

Third, our findings suggest the possibility of controlling abnormal network recoveries by modulating ES proximity. In previous modeling, we demonstrated that network modulation, specifically enhancing hub connections, converts the type of phase transition in the brain network from ES to non-ES, reducing sensitivity to external stimuli (42). Another study showed that adding or removing a few key links can induce abrupt transitions, called “ES bombs,” supporting the potential of modulating ES proximity through local structural changes (43). This line of research could advance novel network modulation methods with the potential, for example, to reduce hypersensitivity in the brain in chronic pain, facilitate the recovery of normal brain functions in pathologic states, and accelerate market recovery after an economic crisis.

Fourth, in our model, we used the peak PCF to identify critical points in the network. Although this approach has been used in previous studies, it has not been validated against other widely accepted criticality indicators. To address this, we rigorously tested whether the peak PCF in our model, as well as in conscious brain states and precrisis financial periods, exhibits typical properties of criticality: scale invariance, long-range temporal correlations, power-law scaling of avalanche size and duration, branching ratio of 1, and rescaling behavior. These were assessed using finite size scaling analysis, detrended fluctuation analysis, the deviation from criticality coefficient, and branching ratio. Our results confirmed that they all exhibit typical features of critical systems, supporting our argument that the ES proximity (the kurtosis of PCF and ACF) measured “at critical states” accounts for gradual and abrupt state transitions during crises. Detailed validation results are provided in *SI Appendix*, Section S4: *Validation of Criticality*.

Additionally, we investigated whether deviations from the criticality coefficient and the Hurst exponent could predict patterns of criticality loss and recovery. If distance from criticality were the primary factor driving gradual or abrupt system collapse and recovery, we would expect these metrics to exhibit predictive power comparable to ES proximity, as quantified by kurtosis of ACF/PCF. However, our results showed that neither deviation from criticality coefficient nor the Hurst exponent correlated with the time to losing or regaining criticality across models, EEG data, or financial networks (*SI Appendix,* Figs. S12–S15). These findings support our central claim: The type of phase transition, as captured by ES proximity, plays a distinct and mechanistically meaningful role in shaping system resilience, beyond what can be explained by criticality distance alone.

### Limitations.

This study has several limitations. First, the observed relationship between signal characteristics (kurtosis of ACF) at critical points, ES proximity, and critical state transitions lacks analytical justification. Future research should aim to develop a mathematical framework to better understand this relationship. Second, given the complexity of the study, we employed a simplified perturbation model to explore the influence of ES proximity on criticality loss and recovery rates. For feasibility, we assumed a uniform perturbation across all networks, because accounting for diverse perturbation forms was beyond the scope of this work. Third, although the Stuart–Landau model is a canonical framework for studying network dynamics near and far from criticality, and can be reduced to the Kuramoto model under certain conditions, it remains to be clarified whether our simulation results generalize to more complex models, such as neural mass models like Wilson–Cowan.

## Conclusion

Our study demonstrates that a network’s proximity to ES at its critical point plays a pivotal role in its resilience to external disruptions. Second-order networks close to the ES limit lose their critical states more rapidly and recover more slowly, reflecting greater susceptibility to perturbation and stronger internal resistance to recovery. These findings pave the way for developing network-specific methods to predict and modulate network collapse and recovery dynamics. This approach has broad applications across complex systems, including brain and financial networks, offering mechanism-based strategies to enhance resilience and prevent or mitigate abnormal transitions during crises.

## Supplementary Material

Appendix 01 (PDF)

## Data Availability

All relevant analysis code, processed data (e.g., ES proximity matrices and derived time series), and representative sample data from both the EEG and stock datasets have been deposited on Github (44). This work utilized previously published human EEG data from an earlier study (45).
