# Supplementary material for: Proximity to explosive synchronization determines network collapse and recovery trajectories in neural and economic crises
Source: Proc Natl Acad Sci U S A. 2025 Oct 30;122(44):e2505434122. doi: 10.1073/pnas.2505434122 (PMC12595435; doi:10.1073/pnas.2505434122)
Supplement: Supplementary file 1 — Appendix 01 (PDF) [file pnas.2505434122.sapp.pdf]

PNAS

Supplementary information for

Proximity to Explosive Synchronization Determines Network Collapse and Recovery Trajectories in Neural and Economic Crises

Authors:

UnCheol Lee[†*1,2], Hyoungkyu Kim[†1], Minkyung Kim[†1], Gabjin Oh[†3], Pangyu Joo[†1], Ayoung Park[†3], Dinesh Pal[1,2], Irene Tracey[4], Catherine E. Warnaby[4], Jamie Sleigh[5], George A. Mashour[1,2]

Affiliations:

1. Department of Anesthesiology, University of Michigan Medical School, Ann Arbor, Michigan, USA
2. Center for Consciousness Science, Michigan Psychedelic Center, Neuroscience Graduate Program, University of Michigan, Ann Arbor, Michigan, USA
3. Division of Business Administration, College of Business, Chosun University, Gwangju, Republic of Korea
4. Wellcome Centre for Integrative Neuroimaging, Oxford Centre for Functional MRI of the Brain (FMRIB), Nuffield Department of Clinical Neurosciences, Nuffield Division of Anaesthetics, University of Oxford, Oxford, United Kingdom
5. Department of Anesthesiology, Faculty of Medical and Health Sciences, University of Auckland, Auckland, New Zealand

[†] The authors contribute to the study equally as the first author.
Hyoungkyu Kim's current affiliations: Research Institute of Slowave Inc., Seoul; Center for Neuroscience Imaging Research, Institute for Basic Science (IBS), Suwon, Republic of Korea
Ayoung Park's current affiliations: Financial Market Stabilization Department, Financial Supervisory Service, Seoul, Republic of Korea

Corresponding Author:
UnCheol Lee
Email: uclee@med.umich.edu

This PDF file includes:

- Supporting text
- Figures S1 to S16
- Tables S1 to S10
- SI References

Supporting Information Text

Contents

S1. Computational Model

S1.1. Modified Stuart-Landau model with adaptive feedback and stimulation terms.

S1.2. Changes in Kurtosis of Autocorrelation Function (KACF) and Kurtosis of Pair Correlation Function (KPCF) with varying adaptive feedback process strength (Z) (Figure S1, Tables S1, S2).

S1.3. Robustness tests with different stimulation strengths and durations (Figure S2).

S1.4. Robustness tests with different network topologies (Figure S3).

S1.5. Robustness of the correlations between KACF and the times to criticality loss and recovery against network sizes (Figure S4)

S2. Human Brain Networks during Anesthesia

S2.1. EEG experimental procedures.

S2.2. EEG data preprocessing, KACF calculation, and statistical analysis.

S3. Stock Market Networks during Economic Crisis

S3.1. Construction of stock market data (Table S3).

S3.2. Stock data preprocessing, KACF and KPCF calculations, and statistical analysis.

S3.3. Robustness tests with varying baseline periods (Table S4).

S3.4. Robustness tests with varying time periods (α) (Table S5).

S3.5. Association of GDP per capita with ES proximity of stock market network (Table S6).

S4. Validation of Criticality

S4.1. Criticality test methods employed:

- Finite-size scaling (FSS), deviation from criticality coefficient (DCC), branching ratio, rescaling analysis, and detrended fluctuation analysis (DFA)

S4.2. Modified Stuart-Landau model data at maximal PCF

- The results of FSS analysis (Figure S5)
- The results of DCC, branching ratio, and rescaling analyses (Figure S6).
- The results of DFA (Figures S7, S8).

S4.3. Human EEG: Conscious state before anesthesia

- The results of DCC (Figure S9, Tables S7, S8).
- The results of DFA (Figure S10).

S4.4. Stock Data: Pre-crisis period

- The results of FSS (Figure S11, Table S9)
- The results of DFA (Figure S12).

S5. Test Whether the Deviation from Criticality in The Pre-Crisis Can Explain Criticality Loss and Recovery Times.

S5.1. Model data

- DCC and Hurst exponent vs. Criticality loss/recovery time (Figure S13)

S5.2. Human brain EEG

- DCC vs Consciousness loss/recovery time (Figure S14).
- Hurst exponent of conscious state vs Consciousness loss/recovery time (Figure S15).

S5.3. Stock data

- Hurst exponent in pre-crisis period vs Market collapse/recovery time (Figure S16, Table S10).

S1. Computational Model

S.1.1. Modified Stuart-Landau Model with Adaptive Feedback and Stimulation Terms:

A large-scale brain network model using coupled Stuart-Landau oscillators at criticality has successfully replicated the brain dynamics in a conscious resting state observed empirically with fMRI, MEG, and EEG[1-3]. Building on the approach, we developed a modified Stuart-Landau model to simulate large variances in state transitions, including the hysteresis phenomenon observed during general anesthesia[4]. These transitions are modulated at critical points, with varying degrees of Explosive Synchronization (ES) proximity. With the addition of a new stimulation term, we examined the relationship between the ES proximity, the loss and recovery of critical state under external perturbation, and the influence of network topology.

The adaptive feedback term R_j^Z represents a recursive interaction process between a node and the other nodes in (S1):

$$\dot{z}_j(t) = \left\{\lambda_j + i\omega_j - \left|z_j(t)\right|^2\right\} z_j(t) + R_j^Z S \sum_{k=1}^{N} A_{jk} K_{jk} z_j\left(t - \tau_{jk}\right) + \beta\xi_j(t)),$$

$$j = 1,2, \dots, N \quad \text{(S1)}$$

Here, the state of the j_{th} oscillator is determined by a complex variable $z_j = r_j\, e^{i\theta_j}$ at time *t*, where $|z_j|^2 = r_j^2$ and r_j and θ_j are the amplitude and phase variables of the oscillator *j* at time *t*, respectively. ω_j is the natural frequency; λ_j controls the decay rate of the amplitude; S is a coupling strength among the oscillators; and A_{jk} is the anatomical connection between node j and k. $\xi_j(t)$ is a Gaussian white noise for each node j and added to the dynamics with the standard deviation β=0.05. The connection matrix A varies with the topologies of the network models used (random, scale-free, small-world, and brain networks). For the brain network, it consists of 78 cortical regions constructed from average diffusion tensor imaging (DTI) of 80 young adults[5]. We set $A_{jk} = 1$, if a connection exists between node j and k, and $A_{jk} = 0$ otherwise. τ_{jk} is a time delay between node j and k**,** which is crucial in the human brain network model.

R_j is defined as:

$$R_j = \frac{1}{2}\left(e^{i\theta_j} + \frac{1}{N}\sum_{K=1}^{N} e^{i\theta_k}\right) \qquad \text{(S2)}$$

which represents the extent of the phase synchrony of node j with the other nodes.

The Control Parameter of ES Proximity Z:

Z is a scale term for the feedback process between one node and its connected nodes. Since R_j is less than 1, a larger Z suppresses the interaction term with a smaller R_j^Z in (S1) and pushes the network's phase transition type closer to ES. R_j^Z with a larger *Z* inhibits the merging of small synchronization clusters, delaying the formation of a giant synchronization cluster in a network. This delayed transition triggers an abrupt synchronization transition at a critical point[6].

Model Parameter Setting:

- Natural Frequencies (ω_j): Gaussian distribution around 10 Hz with a standard deviation of 0.4 Hz.
- Decay Rate of Amplitude (λ_j): Set to 1 for all oscillators.
- Time Delay (τ_{jk}): Proportional to the physical distances between nodes *j* and *k* with a propagation speed of 7 m/s[7].
- Coupling Strength (S): Adiabatically increased and decreased from 0 to 4 with a step size of 0.05.
- Adaptive Feedback Strength (Z): Set to {0,0.5,1,1.5,2,2.5,3} to simulate distant and close ES proximities in the network.

We numerically solved the differential equations using the Stratonovich-Heun method with 1,000 discretization steps. One hundred different initial frequency configurations were simulated for each parameter set, and the results were averaged over all configurations.

Identification of Critical Points in Network Models:

An increase in signal variance and autocorrelation function is characteristic of a system approaching a critical transition, a phenomenon known as *critical slowing down*, where the system takes longer to return to equilibrium after a disturbance[8]. The variance (PCF) and the autocorrelation function (ACF) of the order parameters also reach their maximum at a critical point[9,10,11]. We explored the parameter space to identify the coupling strength that maximizes the PCF of the order parameters, considering the peak as a critical point. We examined the network dynamics characteristics at the critical points for 'distant' and 'close' ES proximities (Z) and studied their distinct response behaviors to external perturbation.

Notably, in this study, we focused on criticality transitions in networks with either 'distant' or 'close' proximity to ES, rather than in networks directly exhibiting ES, as ES rarely occurs in real-world networks under normal conditions. This implies that most of the networks we examined undergo second-order transitions rather than first-order phase transitions. When simulating networks with 'close' ES proximity (e.g., Z=3), the discrete nature of first-order transitions made it challenging to pinpoint the exact coupling strength that produces ES. Therefore, for a first-order like transition, we defined the critical point as the coupling strength just before the discrete transition and evaluated ES in the time domain.

Dynamic noise introduced in the model allows a network in an incoherent state just before ES to cross the critical point, enabling observation of a discrete transition from incoherence to a highly synchronized state in the time domain (Figure S1. D). This transition is marked by significant changes in network dynamics, reflected in extreme ACF and PCF values and an increase in kurtosis in ACF and PCF values. For consistency, we defined the critical point in non-ES networks as the coupling strength just before the PCF peak. Given the fine binning of coupling strengths, the KACF and KPCF values in non-ES networks closely match those observed at the ACF or PCF peak.

Varying ES Proximity and Time to Critical State Loss/Recovery Under Perturbation:

We directly measured the speed of state transitions against an external perturbation by inducing a global pulsatile stimulus to the network and quantifying the change in network dynamics at critical points. The global pulsatile stimulus *u(t)* was applied as follows:

$$\dot{z}_j(t) = \left\{\lambda_j + i\omega_j - \left|z_j(t)\right|^2\right\} z_j(t) + R_j^Z S \sum_{k=1}^{N} A_{jk} K_{jk} z_j\left(t - \tau_{jk}\right) + \beta \xi_j(t) + u(t),$$

$$j = 1,2, \dots, N. \quad \text{(S3)}$$

$$u(t) = \begin{cases} p, & t_1 < t < t_2 \\ 0, & otherwise \end{cases}$$

where p is the intensity of the stimulus during the period $T = t_2 - t_1$. We tested various stimulus strengths p = 10, 20, 40, 60, 80, 100, and durations T = 0.1, 0.2, 0.5,1, 2, 5, 10, and 20 seconds, which are strong and long enough to influence the transition patterns during stimulation[10]. The global stimulus was applied at random timings t_1 for each trial. We conducted 20 trials for each of the 100 initial frequency configurations, resulting in 2,000 stimulus applications across the network at critical points.

The time to lose the critical state was defined as the time from stimulus onset until the order parameter exceeded three times the pre-stimulus standard deviation (at critical points). The time to recovery was defined as the time after stimulus cessation until the order parameter returned below three times the pre-stimulus standard deviation.

Order Parameter: The complex order parameter at time *t* is defined as:

$$z\,(t) = r(t)e^{i\psi(t)} = \frac{1}{N}\sum_{f=1}^{N} e^{i\theta(j,t)}, \qquad \text{(S4)}$$

Where $z\,(t)$ is a complex order parameter at time t. $\psi(t)$ is the average phase over multivariate time series signals at a time *t*; and $\theta(j,t)$ is the phase of a signal j at a time *t*. The absolute value $r(t) = |z\,(t)\,|$ is the instantaneous order parameter at a time *t*, which represents the average phase synchronization over all individual signals at time t. $r(t)$ equals 1 when all signals are fully synchronized with the same instantaneous phase and 0 when the signals are completely incoherent, with the instantaneous phases uniformly distributed in $[0,2\pi)$ at time *t*.

Calculation of Kurtoses of ACF values (KACF) and PCF values (KPCF):

The KACF/KPCF was calculated using the multivariate signals generated at a critical point as follows:

1. Extract instantaneous phases by applying the Hilbert transform to multivariate signals.
2. Calculate the instantaneous order parameters, defined in the coupled Kuramoto oscillators model.
3. Apply the moving window method to the instantaneous order parameters.
4. Calculate the autocorrelation function (ACF) and the pair correlation function (PCF) for each window

5. Calculate the kurtoses of ACFs and PCFs over the windows.

Autocorrelation Function (ACF) and Pair Correlation Function (PCF) of Order Parameters:

For a given data, we applied the moving window method with a window size that satisfies the pseudo-stationarity of dynamics, moving with a half-size window overlap. For each data in this study, the simulated signals, EEG signals, and daily stock returns, set an appropriate window size. The $ACF_k(\tau)$ of the order parameter $r(t)$ for a window k is calculated for lag τ as:

$$ACF_k(\tau) = \frac{\sum_{t=1}^{N-\tau} (r(t)-\mu_k)(r(t+\tau)-\mu_k)}{\sum_{t=1}^{N} (r(t)-\mu_k)^2} \quad \text{(S5)}$$

Where μ_k is the mean of $r(t)$ within window k, and N is the total number of the order parameter samples in a window k. An appropriate time lag τ was selected for each type of signal (simulation, EEG, and stock price), and the robustness of its selection was tested with several time lags.

Finally, for a given sequence of $ACF_k(\tau)$, k =1, …, M, where M is the total number of windows. The sample kurtosis is calculated using:

$$Kurtosis\ of\ ACF = \frac{N \sum_{k=1}^{M} (ACF_k-\mu_{ACF})^4}{(\sum_{k=1}^{M} (ACF_k-\mu_{ACF})^2)^2} \quad \text{(S6)}$$

where μ_{ACF} represents the mean of the sequence of ACFs over all windows. The kurtosis of the ACFs measures whether the ACFs of order parameters *r(t)* are heavy-tailed compared to the normal distribution. We expect that a network with close ES proximity likely exhibits typical signal properties of a first-order phase transition near a critical point, such as intermittent and bistable transition dynamics, which are observed as outliers, compared to a network with distant ES proximity.

In addition, the Pair Correlation Function (PCF)[11] was calculated using:

$$PCF = \langle Re^2[z(t)]\rangle_t - \langle Re[z(t)]\rangle_t^2 \quad \text{(S7)}$$

where $Re\,(z(t))$ denotes the real part of the complex order parameter at time t. The kurtosis of PCF was calculated using the same process as for ACF, with PCF replacing ACF. We found that the kurtosis of PCF (KPCF) is significantly correlated with the times of critical state loss and recovery in daily stock prices during crises. However, we couldn't find significant correlations in human EEG during anesthesia. Therefore, we presented the KACF results in the main text.

S1.2. Changes in Kurtosis of Autocorrelation Function (KACF) and Kurtosis of Pair Correlation Function (KPCF) with varying adaptive feedback process strength (Z). For Z=0 (Figure S1.A), the network exhibits typical dynamics near and far from the critical point. As the network approaches the critical points, the variance of instantaneous order parameters gradually increases, reflected by increasing PDF values. Based on previous studies[2,3,4,10,11], we defined the maximum PCF as the critical point. Across networks with varying ES proximities (from Z=0 to 3), the increase in PCF up to the critical points remains consistent. As Z increases (e.g., Z=1 and 2, shown in Figure S1.B and C), the network

began to display intermittent state transitions at critical points, alternating between extended periods of highly synchronized state and desynchronized state. At Z=3, the network dynamics become sharply bistable, shifting abruptly to a highly synchronized state at the critical point.

Calculation of KACF and KPCF and Statistical Analysis: The temporal dynamics of the order parameter vary with different Z values. Time series signals were generated at critical points, with 300 seconds of data collected after an initial transition period. Using a moving window method (10-second window, 5-second overlap), we calculated the ACF and PCF of instantaneous order parameters within each window. For each Z value, KACF and KPCF were calculated from 59 ACF and 59 PCF values, respectively. Each simulation was repeated 100 times with random initial conditions. The mean and standard error of KACF and KPCF at critical points are shown in Figure S1 (E) and (F), respectively. The results indicate that as network's phase transition type approaches ES, the KACF and KPCF of network dynamics at critical points significantly increase, supporting both indexes as indicators of ES proximity. Statistical analysis, conducted via one-way ANOVA with post-hoc LSC and Tukey-Kramer tests, further validates these findings.

S1.3. Robustness Tests with Different Stimulation Parameters: To ensure the robustness of our findings, we examined how different stimulation parameters influence the relationship between ES proximity and network stability. Figure S2 illustrates the results of these tests, where we varied both the stimulation strength (u) and duration. The analysis confirms that the primary relationship holds: networks with closer proximity to ES (larger Z) consistently demonstrate a faster loss of criticality but a slower recovery. This pattern remains consistent across a wide range of stimulation strengths (u = 20, 40, 60, 80, 100) and durations (from 0.1 to 20 seconds). Notably, the relationship becomes most pronounced for stimulation strengths sufficient to push the network away from its baseline critical state (u > 20). These results demonstrate that the qualitative relationship between ES proximity and stability is robust to the external perturbation.

S1.4. Robustness Tests with Different Network Topologies: We also tested the robustness of the simulation results using different network topologies, including random, scale-free, and small-world networks. Figure S3 shows how ES proximity (Z) relates to network topology and the time to critical state loss and recovery. Networks with closer ES proximity (larger Z) exhibit faster critical state loss and slower recovery, compared to networks with distant ES proximity (smaller Z). This trend is observed across all network topologies: (A) Small-world, (B) Random, and (C) Scale-free networks. Each network comprises 1,000 coupled Stuart-Landau oscillators (nodes) with an average node degree of 2. Error bars represent the standard errors from 100 simulations, each with different initial conditions. Both the small-world (Watts-Strogatz) network[12] and random (Erdős-Rényi) network[13] show higher sensitivity to changes in Z, with notable differences in the time to critical state loss and recovery. In contrast, the scale-free (Barabási-Albert) network[14] displays less sensitivity to variations in Z. This differential sensitivity is due to the characteristic topologies of these networks. The high clustering in small-world networks and the heterogeneity in random networks may enhance conditions for ES, inhibiting the formation of a giant synchronized cluster even at the same feedback strength (Z). On the other hand, the presence of highly connected hubs in scale-free networks may facilitate smoother phase transitions, reducing the likelihood of delayed transitions. These results suggest that while the relationship between ES proximity and critical state loss and

recovery is maintained across different topologies, the nature of the phase transition at the critical state is influenced by network topology.

S1.5. Robustness of the correlations between KACF and the times to criticality loss and recovery against network sizes. To test the effect of network size and ensure our findings were not dependent on the specific size of the 78-node brain network, we performed additional simulations on random networks of varying sizes (N = 25, 50, 75, 100, 150, 250, and 500). The average node degree was kept consistent with the original network to ensure comparability. We then assessed the correlation between our ES proximity measure, KACF, and the times to lose and regain criticality under perturbation for each network size. As shown in Figure S4, the fundamental relationships remained robust across all tested sizes. The time to lose criticality consistently showed a strong negative correlation with KACF, while the time to regain criticality maintained a strong positive correlation. Although minor fluctuations in the correlation coefficients were observed, no systematic trend was evident, indicating that the observed effects did not diminish or disappear as the network size increased. This analysis confirms that our conclusions are not an artifact of the specific network size used and that the relationship between ES proximity and network stability is a general feature of the model dynamics.

S2. Human Brain Networks during Anesthesia

S2.1. EEG Experimental Procedures: We utilized 32-channel EEG data from 16 healthy subjects (8 male, 8 female; age 28.6±7.028.6±7.0 years; range 19–43 years) participating in a study involving an ultraslow target-controlled intravenous infusion of propofol, a γ-aminobutyric acid type A ($GABA_A$) agonist. The experiment was designed to observe brain dynamics during the induction of and emergence from deep sedation induced by propofol[15]. The central nervous system propofol effect-site concentration (ESC) was estimated throughout the experiment.

The EEG data comprised four distinct phases:

1. **Baseline Resting State**: 10 minutes of eyes-closed resting state to establish baseline measurements.
2. **Induction Phase**: Ultraslow propofol-induced loss of consciousness over 48 minutes, during which the estimated ESC gradually increased to a maximum of 4 μg/ml.
3. **Maintenance Phase**: A 10-minute period maintaining the peak propofol dose to observe sustained effects on brain activity.
4. **Recovery Phase**: Observation of brain activity during the recovery of consciousness after the propofol infusion was discontinued.

During phases 2 and 4, laser stimuli, auditory stimuli, and cognitive word tasks were randomly delivered at 1-minute intervals to assess sensory processing and cognitive function. The Loss of Behavioral Response (LOBR) was defined as the time point when a subject ceased responding to the word task, indicating a transition into unconsciousness. Conversely, the Recovery of Behavioral Response (ROBR) was marked by the time point when the subject resumed responding, indicating a return to consciousness. These data have been previously analyzed and published in separate studies[15,16]. Detailed experimental designs and protocols are thoroughly described in these prior publications.

S2.2. EEG Preprocessing: EEG data were re-referenced using the average reference method and downsampled to 125Hz from 500Hz. A windowed sinc-FIR filter (EEGLAB MATLAB toolbox) was applied to prevent phase shifting in the signals. A bandpass filter was applied to isolate the alpha-frequency band (8–13 Hz). The Hilbert transform was then used to extract instantaneous phases from 31-channel EEG signals (excluding one reference channel) and calculated the instantaneous order parameters using (S4).

KACF Calculation: The moving window method was employed with a window size of 10 seconds and a 5-second overlap between windows. ACF values were calculated for each window using a time lag of 11, reflecting the dynamics of the alpha band, ~10Hz (as in equation S4). The KACF was computed from the ACF values across all windows.

Statistical Analysis: The Spearman correlation coefficient was calculated between the KACF values and the induction and recovery times of 16 subjects.

S3. Stock Market Networks during Economic Crisis

This supplementary information on economic data analysis provides further details on the methods used to study financial market crises in 39 countries. It outlines the construction of the financial data sample, including the selection criteria for firms in each country, and provides a detailed explanation of the data analysis procedures. The section also addresses the robustness of the results, focusing on the choice of the baseline period and the time window used to identify market collapse and recovery during the economic crisis. Lastly, it explores the relationship between GDP per capita and the ES proximity of financial networks.

S3.1. Construction of Stock Market Data: Financial market data for this study were sourced from S&P Compustat Global and the Center for Research in Security Prices (CRSP) databases. These comprehensive databases provide detailed information on a wide range of global firms, including daily stock prices, shares outstanding, total assets, trading volume, exchange codes, and share codes. The number of firms analyzed varied between 11 and 2,664, depending on the country. This dataset provided the foundation for examining the economic impact of the 2008 global financial crisis across different countries.

The countries included were selected based on the availability of high-quality stock market data, representing a mix of developed and emerging economies to capture a wide range of market dynamics. Table S3 provides key details such as the representative stock market index for each country, the number of companies included, recession periods, and GDP per capita. For countries without OECD recession indicators (e.g., Thailand, Pakistan, Malaysia), the U.S. recession period was used as a reference.

Data Selection Criteria:

- **Firm-Level Data**: We focused on common stocks (share codes 10 and 11), which typically represent ordinary shares listed on the primary exchange in each country. Companies that reported negative total assets at any time during the analysis period were excluded to ensure data integrity. In cases where multiple share classes existed, the common share class with the highest total stock assets was chosen for consistency.

- **Country-Level Criteria**: Countries with insufficient data or irregular recession periods were excluded. For instance, Denmark was excluded because its recession began too early (July 2006), while Greece was excluded because its economic recovery extended beyond the study's timeframe (until 2011). After these exclusions, 39 countries remained, categorized as either developed or emerging markets, allowing for comparative analysis across different economic contexts.

Dataset Overview: The dataset was carefully curated to include diverse economies and geographic regions, aiming to analyze the heterogeneous impact of the 2008 financial crisis. Countries were selected based on the availability of sufficient stock data, focusing on both developed and emerging economies to represent different stages of economic development.

- **Stock Data Sources**: We collected daily stock data from the main exchanges in each country. In cases where a country had multiple significant exchanges, data from all relevant exchanges were included to ensure a representative sample size.
- **U.S. Companies**: Classified using the Permno code from the Center for Research in Security Prices (CRSP) database. The focus was on companies listed on the New York Stock Exchange (NYSE) and the American Stock Exchange (AMEX).
- **Other Countries**:
  - For South Korea, both KOSPI and KOSDAQ were included.
  - For India, companies from both the National Stock Exchange (NSE) and the Bombay Stock Exchange (BSE) were considered.
  - For Russia, we included companies from the Moscow Exchange (MOEX), the Russian Trading System (RTS), and the Saint Petersburg Exchange.
  - For China, firms listed on the Shanghai Stock Exchange (SSE) and the Shenzhen Stock Exchange (SZSE) were analyzed.
  - For Taiwan, companies listed on both the Taiwan Stock Exchange (TWSE) and the Taipei Exchange (TPEx) were included.

Pre-Crisis Normal Period: The "normal period" was defined as January 2006 to December 2006, chosen as a stable phase before the onset of the 2007-2009 global financial crisis. This period served as a baseline to evaluate the ES proximity of each country's stock market under normal, pre-crisis conditions.

- **Data Cleaning**: During the sample period from July 2005 to December 2006, we removed companies with more than 95% zero values for their stock prices. This ensured that only actively traded firms were included, enhancing the reliability of the analysis.
- **ES proximity Measure**: To estimate KACF, we used a window size of 120 days. Figures 5C and 5D in the main text present the Spearman correlations between the KACF and response and recovery times, demonstrating the linkage between

market conditions during the pre-crisis period and subsequent market performance during the crisis.

- **Sensitivity Analysis**: To ensure the robustness of our findings, we conducted a sensitivity analysis by systematically varying the start point of the pre-crisis period. We adjusted the starting point within a six-month window (from July 2005 to January 2006) and recalculated the ES proximity (KACF) for each country. The results were consistent, confirming the significant relationship between ES proximity across different pre-crisis periods and market response/recovery times during the crisis (as shown in Table S4).

S3.2. Stock data preprocessing, KACF and KPCF calculations, and statistical analysis. In this study, we define a country's market collapse and recovery time during an economic crisis using changes in the stock market index (e.g., S&P500 index), while individual firm stock prices (e.g., Apple Inc.) are utilized to calculate market dynamics such as ES proximity or market criticality. A stock market index is a statistical measure reflecting the overall performance of a specific group of stocks (e.g., the S&P 500 index, which tracks 500 large US companies; the Nasdaq, focused on technology stocks). The stock market index P_c^m of a country c and the stock price P_c^f of a firm f are analogous to anesthetic concentration and a single-channel EEG signal in the brain, respectively.

Definition of response time and recovery time: The stock return $R_c^f(t)$ of a firm (*f*) in 39 countries (*c*) is computed as the difference of the natural logarithms of adjusted stock prices.

$$R_c^f(t) = log\ (aP_c^f(t)) - log\ (aP_c^f(t-1)\ ),\ \ \{f = 1,2,\dots,N_c\} \in SC_c$$

$$aP_c^f(t) = \left(\frac{P_c^f(t)}{ajexdi^f(t)}\right) \times Trfd^f(t) \qquad \text{(S8)}$$

Where SC_c means the set of available firms *f* in a country c, and t represents the daily time frequency from July 2005 to December 2006. To measure the stock returns $R_c^f(t)$, we first calculated the adjusted stock prices $aP_c^f(i,t)$ for all available firms of each country by using the information about $P_c^f(i,t)$, $ajexdi(i,t)$, and $Trfd(i,t)$ from S&P Compustat Global. $P_c^f(t)$ means the closing stock price of a firm f listed in a major exchange of a country c. $ajexdi(i,t)$ is an adjusted factor for all stock splits and dividends, providing a more accurate reflection of a company's equity value over time by removing the effects of these corporate actions of the firm i at a time t. Trfd is a daily factor that captures the total return of a stock, encompassing both price appreciation and dividend reinvestment, providing a comprehensive measure of investment performance. This price adjustment ensures that the stock returns reflect the effects of corporate actions, such as stock splits, mergers, acquisitions, and dividends.

We tested whether the KACF measured during the normal period correlates with each country's response and recovery time in the 2008 economic crisis. To account for differences in stock market scales across 39 countries, we normalized stock market index changes during the crisis and defined market collapse and recovery rates based on the extent of each country's price drop, allowing direct comparisons. Response rate (R1) and recovery rate (R2) were measured using equation (S9).

$$R1(c) = \frac{P_c^m(t_1) - P_c^m(t_1+\alpha)}{P_c^m(t_1) - P_c^m(t_2)}, \; R2(c) = \frac{P_c^m(t_2+\alpha) - P_c^m(t_2)}{P_c^m(t_1) - P_c^m(t_2)} \quad \text{(S9)}$$

Where $P_c^m(t)$ is the stock market index of the country c and time $t = 1, 2, 3, \ldots T$. $t_1 = \{t \in T : \max P_c^m(t)\}$ and $t_2 = \{t \in T : \min P_c^m(t)\}$, indicate the dates of the maximal and minimal stock market index in the country c, respectively. These rates mean the price difference between the maximum (minimum) price and a reference point, which is how much the stock market index decreases(increases) from the maximum (minimum) prices for a given time α (illustrated in Figure 5B). We assumed that the maximum and minimum prices for each country during the 2008 crisis indicate, respectively, the turning points at which a market enters and exits its crisis.

We set the reference point as the price after α days from the maximum (or minimum) price. Because the 39 countries show diverse market dynamics, we tested several time periods $\alpha = \{40, 60, 80, 100, \; and \; 120 \; days\}$. 40 and 120 days approximately correspond to 2 and 6 months, considering market closing days. We chose α of 100 days, an appropriate period to reflect the temporal scale of the overall price changes during the worldwide economic crisis. The sensitivity of the α selection was tested in Tables S4 and S5. Finally, we applied a logarithm to the inverse of the market response and recovery rates for direct comparison with the EEG study's induction time and recovery time.

$$Response\ time(c) = \ln\left(\frac{1}{R1(c)}\right),\ Recovery\ time(c) = \ln\left(\frac{1}{R2(c)}\right), \quad \text{(S10)}$$

If the country has a higher response (recovery) rate, it represents a fast financial market collapse (recovery). We anticipate the country's stock market will be healthy if it has a longer response time and a shorter recovery time.

Indicators of ES proximity in a stock market: KACF and KPCF. In this section, we investigate the hypothesis that the ES proximity of a stock market network influences its resilience and recovery capabilities during the 2008 economic crisis. Our analysis focuses on the relationship between the ES proximity and the time to respond to and recover from the crisis. Using the same procedure as in the EEG study, we computed the KACF as follows: (1) extract the phases of stock returns, (2) calculate the ACFs of the moving window, (3) calculate the KACF over all the windows.

1. Apply the Hilbert transform to the daily stock returns of the normal period from July 1, 2005, to December 31, 2006, and obtain the instantaneous phases for each firm f in country c.
2. Calculate the instantaneous order parameters using the multivariate phases of the listed firms for each country c, as specified in S4.
3. Calculate the ACF of each window (window size of 120 days, 1-day overlapping) using (S5), which produces approximately 250 ACF values during the normal period for each country. We tested different time lags (τ = 1, 2, and 3) and presented the robustness of the τ t selection in Table S5. In the main text, we present the result of τ= 2.
4. Calculate the KACF over the normal period for a county using (S6). Repeated this procedure (1), (2), and (3) for 39 countries.

In addition, we tested ES proximity using an alternative criticality indicator, Pair Correlation Function (PCF). We repeated the procedure (1)-(3) after replacing ACF with PCF, defined

in (S7). We demonstrated that the kurtosis of PCF (KPCF) during the normal period has significant correlations with both the response time and the recovery time, as shown in Table S6.

Notably, despite KACF showing correlations in all the data (simulated data, EEG, and stock price), we did not find a significant correlation with KPCF in the EEG study. This might be influenced by the relatively small number of channels (31 channels). Considering that the order parameter measures the global phase coherence in a network, a small number of samples may be insufficient to accurately reflect the global network dynamics of the brain during anesthesia.

S3.3 and S3.4. Robustness to the varying normal periods, alpha α, and time lag: In Table S5, we evaluated how the selection of the "normal period" influences the Spearman correlations between response/recovery times and the KACF. The normal period was defined based on the principles of the Efficient Market Hypothesis (EMH) to ensure it represents a stable phase before the onset of the subprime financial crisis. We tested three different normal periods and nine values of alpha (α), which represent the duration after the market's minimum (or maximum) point, used to calculate both response and recovery times. Our results indicate that the choice of the normal period does not significantly affect the correlation between market response/recovery times and the KACF.

In Table S5, we tested the robustness of the KACF correlation by varying both the time lags (1, 2, and 3) and the alpha (α) values (ranging from 40 to 120 days). We found that the KACF with a time lag of 2 showed a significant correlation with both response and recovery times. Additionally, testing with KPCF also revealed significant correlations with response/recovery times, but only for alpha (α) of 40 days. This suggests that KPCF is more sensitive to the specific methods used for defining response and recovery times.

S3.5. Association of GDP per capita with ES proximity of stock market network. To test the robustness of the potential relationship between countries' economic development and the stock market's ES proximity, we analyzed Gross Domestic Product (GDP) per capita for 39 countries in 2006, selected as the pre-crisis year (see Table S3). We evaluated two ES proximity indicators - KPCF and KACF at lags 2- using various normal periods. Table S6 shows significant negative Spearman correlations between both ES proximity indicators and GDP per capita. This correlation persisted even when adjusting the normal period. The observed correlations between ES proximities of countries' stock market networks and the levels of economic development may explain why 'developed' countries, with more distant ES proximity, tend to be more resilient during the crisis compared to 'developing' countries, which exhibit closer ES proximity and are more susceptible to phase transitions.

S4. Validation of Criticality

To validate whether the Stuart–Landau model at maximum PCF, EEG in the conscious resting state before anesthesia, and stock markets in the pre-crisis period reflect critical dynamics, we applied several criticality tests: finite-size scaling (FSS), avalanche-based measures (Deviation from Criticality Coefficient and Branching Ratio), and detrended fluctuation analysis (DFA). For the model data, we systematically compared critical and subcritical states by identifying the coupling strength at which the pair correlation function (PCF) is maximal and defining this as the critical point. Subcritical states were defined as

points where the PCF declined to 75%, 50%, and 25% of its peak value, corresponding to progressively weaker coupling. For each simulation with different initial conditions, the nearest coupling strength to the maximal PCF was selected. Supercritical states were not analyzed because, under explosive synchronization, the network rapidly collapses into full synchrony, making finite-size scaling and avalanche analyses infeasible.

S4.1. Criticality test methods employed

Finite-size scaling (FSS) analysis: In an infinite system at criticality, certain quantities (like correlation length, susceptibility, or cluster size) diverge and follow exact power laws. But in real systems, which are always finite, these divergences are truncated, causing the scaling laws to appear rounded or shifted. Finite-size scaling (FSS) is a standard method in statistical physics to test whether observed scaling reflects true criticality or merely a finite-size artifact[17].

For each network size *N*, we constructed adjacency matrices based on the phase-lag index (PLI) for the Stuart–Landau model and correlation matrices for stock return data. The percolation control parameter p was defined as the density of retained edges after thresholding. For each *N*, the pseudo-critical threshold $p_c^*(N)$ was defined as the edge density at which the susceptibility proxy $\langle s \rangle_{\geq 2}$ is maximized, refined by a local quadratic fit. From the pairwise interaction matrix *C, we* constructed an undirected, unweighted *graph* by retaining the top fraction *p* of off-diagonal interaction strengths (rank-thresholding), yielding adjacency $A_N(p)$. Sweeping *p* plays the role of the percolation control.

Let N_s be the number of *finite* clusters of size *s,* and define the normalized frequency as $n_s := N_s/N$. The giant cluster is excluded throughout. The complementary cumulative distribution function (CCDF) of a cluster size is

$$N_c(\text{s}) = \sum\nolimits'_{s' \geq 1} n_{s'}.$$

Where the prime indicates exclusion of the giant component. The susceptibility proxy (mean finite-cluster size) is

$$\langle s \rangle = \frac{\sum'_{s' \geq 1} s^2 n_s}{\sum'_{s' \geq 1} s n_s}$$

Our baseline analysis further excludes singletons (*islands*, *s* = 1):

$$\langle s \rangle_{\geq 2} = \frac{\sum_{s=2}^{s<s_{max}} s^2 n_s}{\sum_{s \geq 2}^{s<s_{max}} s n_s},$$

The pseudo-critical density $p_c^*(\text{N})$ is taken as the value of *p* that maximizes $\langle s \rangle_{\geq 2}(\text{p})$. At $p = p_c^*$, we also estimate the CCDF central slope and report the Fisher exponent τ from the best-fitting window:

$$\text{N}_\text{c}(\text{s}) \propto \text{s}^{-(\tau-1)} \text{e}^{-\text{s}/\text{s}^*}.$$

Scaling relationship at $p \cong p_c^*(N)$ are**:**

$$\langle s \rangle_{\geq 2}|_{p_c^*} \sim N^{w_2} \;, \;\; \langle N_2 \rangle|_{p_c^*} \sim N^{w_1}.$$

Where $\langle N_2 \rangle$ is the average size of the second-largest cluster. In the standard dictionary,

$$w_1 = d_f/d \text{ and } w_2 = \gamma/(\nu d).$$

Thus, the triplet (ω_1, ω_2, τ) serves as the benchmark for universality classification. Point estimates were obtained by ordinary least squares (OLS) fits of $\log\langle N_2 \rangle$ and $\log\langle w \rangle_{\geq 2}$ against log N over network size $N \in$ K. The fitted slopes are reported in Figure S5 and S6 and Table 9. For the CCDF, the exponent τ is estimated as the OLS slope of $\log N_c(s)$ vs log s within a central stability window.

Theoretical reference values for universality classes are:

- mean-field (MF): $\tau = 2.5$, $w_1 = 2/3$, $w_2 = 1/3$
- 2D percolation: $\tau = 187/91 \approx 2.055$, $w_1 = 91/96 \approx 0.948$, $w_2 = 43/48 \approx 0.896$
- 3D percolation: $\tau \approx 2.15$, $w_1 \approx 0.84$, $w_2 \approx 0.67$

Comparing empirical exponents with these theoretical benchmarks situates each system within a universality class, demonstrating that observed scaling reflects genuine critical behavior rather than arbitrary finite-size effects.

Deviation from criticality coefficient: The Deviation from Criticality Coefficient (DCC) is a quantitative, scalar metric used to assess a system's proximity to a critical state[18]. It is based on the principle that neuronal avalanches in a critical system adhere to specific scaling relationships between their size and duration distributions. The DCC is calculated by comparing a theoretically predicted scaling exponent, $\beta_{pred} = (\alpha - 1)/(\tau - 1)$ — derived from the power-law exponents for avalanche size (τ) and duration (α)—with an empirically measured exponent, β_{fit}, obtained from the relationship between average avalanche size and duration. The final coefficient is the absolute difference between these two values, DCC = $|\beta_{pred} - \beta_{fit}|$. A DCC value approaching zero signifies that the system is operating near criticality, whereas a larger value indicates a departure into a subcritical or supercritical regime.

Branching ratio: In addition, we analyzed the branching ratio σ, a fundamental measure used to characterize the propagation of activity in a network[19]. The branching ratio is defined as the average number of subsequent events (or active nodes) triggered by a single event in the preceding time step. This metric provides a direct test of the criticality hypothesis, where a system with σ = 1 is considered critical, as activity is sustained on average. In contrast, a subcritical system (σ < 1) will have decaying activity, while a supercritical system (σ > 1) will exhibit runaway amplification. Therefore, by computing this ratio, we could directly assess whether the network was operating in the dynamically balanced regime characteristic of criticality. The branching ratio is calculated using NCC toolbox[20], and our analysis of the branching ratio provides converging evidence.

Rescaling analysis: To test for scale invariance using the FSS analysis, a hallmark of critical systems, we investigated whether the DCC and Branching ratio yield consistent results, independent of the network size N. We subsampled the original 78-node connectivity matrix into subnetworks of varying sizes (N=16 to 78), while keeping the feedback strength fixed at Z=0, and examined their DCC values and Branching ratios.

Detrended fluctuation analysis (DFA): Detrended fluctuation analysis (DFA) measures long-range temporal correlations in time series, one of the typical system properties of criticality, by estimating the Hurst exponent[21,22]. For each integrated signal,

$$Y(t) = \sum_{k=1}^{t} (X(k) - \bar{X})$$

The local variance after detrending is

$$F^2(s) = \frac{1}{T}\sum_{t=1}^{T} [Y(t) - Y_{\text{fit}}(t)]^2$$

and the fluctuation function scales as

$$F(s) \propto s^H$$

Here, $H \approx 0.5$ corresponds to uncorrelated (random) dynamics, while $H > 0.5$ indicates persistent long-range correlations typical of critical systems. DFA thus complements structural scaling analyses (such as FSS) by probing temporal correlations. By combining DFA with universality-class comparisons from FSS, we can validate both the spatial and temporal signatures of criticality in the studied networks. For the empirical application to the finite size of daily stock data, we aggregate all component series into a composite signal:

$$X_{\text{agg}}(t) = \sum_{i=1}^{N} X_i\,(t)$$

and applies DFA to $X_{\text{agg}}(t)$. However, this method assumes synchrony across components and ignores potential heterogeneity or phase mismatch, leading to possibly distorted or uninformative Hurst exponent estimates. Let $\{X_i(t)\}_{i=1}^{N}$ be a multivariate time series with N components. Apply second-order DFA to each component $X_i(t)$ to obtain its fluctuation function $F_i(s)$. We define the averaged fluctuation variance as:

$$\bar{F}^2(s) = \frac{1}{N}\sum_{i=1}^{N} F_i^2\,(s)$$

and the corresponding averaged fluctuation function:

$$\bar{F}(s) = \sqrt{\bar{F}^2(s)}$$

We then estimate the representative Hurst exponent by fitting:

$$\bar{F}(s) \propto s^H$$

This method preserves the temporal correlation structure of each component while providing a meaningful aggregate Hurst exponent. Compared to aggregation-based DFA, it is robust to phase shifts, tolerant of heterogeneity, and retains statistical interpretability.

This approach is particularly suited for applications in complex systems such as financial markets, neural data, and coupled oscillatory networks.

S4.2. Modified Stuart-Landau model data at maximal PCF

The results of the FSS analysis: We analyzed the output of the Stuart-Landau oscillator model to probe for signatures of criticality using FSS. The analysis was performed on network ensembles generated at the critical point for each network size, which was identified as the coupling strength that maximizes the PCF. We varied the system size N across a set of discrete values (25, 50, 75, 100, 150, 250, and 500). For each network size, 100 independent iterations were performed, and a weighted network was constructed from the phase lag indices between all oscillator pairs for each iteration. From these functional networks, we performed an FSS analysis. For each network realization, we identified the maximum value of the susceptibility, $< s >_{max}$, and the second-largest cluster, $< N^2 >_{max}$, across a sweep of percolation densities. These peak values were then used for the FSS analysis. The CCDF and its corresponding slope were specifically calculated at the pseudo-critical density defined by the peak of susceptibility. The figure S5 summarizes the finite-size scaling readouts for the model. The CCDF of cluster sizes exhibits a central slope of approximately 0.8, implying a Fisher exponent $\tau \approx 1.8$. Concurrently, the size-scaling of the second-largest component and the susceptibility proxy yields $\hat{\omega}_1 = 0.990$ and $\hat{\omega}_2 = 0.909$, respectively. This set of exponents is broadly consistent with a 2D percolation signature; the theoretical values for the 2D percolation universality class are $\tau = 187/91 \approx 2.055$, $\hat{\omega}_1 = d_f/d$ (91/96≈0.948), and $\hat{\omega}_2 = \gamma/vd$ (43/48≈0.896). Taken together, these finite-size-scaling signatures provide strong evidence that the modified Stuart-Landau model at maximum PCF operates in a genuine criticality regime, not one dictated by finite-size artifacts.

The results of DCC, branching ratio, and rescaling analyses: Avalanche analysis provides a powerful framework for identifying critical dynamics in neural systems, where cascades of activity are found to follow power-law distributions in size and duration[17]. To apply this established framework to our model, we first adapted the simulation to reliably generate such spontaneous avalanches. The model's original minimal external input used on the main text, with a dynamical noise standard deviation of 1, was insufficient for this analysis. We therefore increased the noise standard deviation to 40, an empirically chosen value that robustly induces avalanches by mimicking ongoing sensory input without overwhelming the network's intrinsic dynamics. To ensure a sufficient number of these avalanches for statistical analysis, we ran 1800-second-long simulations for each iteration. Because the amplitude signal of the Stuart-Landau model follows a non-Gaussian distribution, we defined discrete events by setting a threshold at the 99.5th percentile of the signal's amplitude. For the analysis of these avalanches, we utilized the NCC toolbox developed by Nicholas Timme, but adapted its fitting procedure to align with established best practices for power-law fitting[18]. Specifically, we required the fitting range to span at

least a fivefold interval on a logarithmic scale (e.g., from 10 to 50) to ensure the analysis was performed on a sufficiently broad range of event sizes. From the candidate ranges meeting this criterion, we selected the one that minimized the Kolmogorov-Smirnov (KS) statistic to ensure the most rigorous fit.

A DCC value approaching zero signifies that the system is operating near criticality, whereas a larger value indicates a departure into a subcritical or supercritical regime. As shown in the left panel of Figure S6D, our analysis reveals a clear relationship between the PCF-defined states (representing graded deviations from criticality) and the corresponding DCC values. The DCC value is systematically minimized at 100% of the peak PCF, which we designated as the critical point, and increased while deviating from the peak PCF. This indicates that the scaling relationship between avalanche size and duration aligns most with theoretical predictions for a critical system precisely at the PCF peak. This result was robust across all tested values of the feedback parameter Z.

The right panel of Figure S6D shows that the branching ratio increases monotonically as the system approaches the 100% peak PCF. At this point, the ratio reaches its maximum value, approaching the critical threshold of $\sigma = 1$. This behavior, consistent across all Z conditions tested, confirms that the system with varying Z operates in a criticality regime at the peak PCF.

As demonstrated in Figure S6E, the qualitative relationship between the PCF-defined state and the criticality metrics (DCC and branching ratio) remained consistent across all tested system sizes. For each network scale, the DCC value was consistently minimized, and the branching ratio increasingly approached to 1 at the 100% peak PCF. This result confirms the scale-invariant nature of the observed dynamics and validates that our PCF-based method robustly identifies the critical point regardless of system size.

The results of DFA: Figure S7 (A) illustrates the long-range temporal correlation assessed by detrended fluctuation analysis (DFA). Fluctuation functions $F(s)$ computed from simulated multivariate time series data from a 78-node Stuart–Landau network model across explosive synchronization (ES) proximity values $Z = 0$ to $Z = 3$. The colored dashed lines indicate fitted scaling behavior $F(s) \sim s^H$ over the range $s = 100$ to $s = 500$. All curves follow nearly parallel power-law trajectories in the log-log plot, reflecting a long-range scaling structure across Z. The slope for $Z=0$ yields a Hurst exponent of $H \approx 1.05$, indicating super-diffusive behavior and strong temporal persistence. The robustness of the scaling exponent across Z suggests that temporal correlations remain intact as the system approaches the ES transition, supporting the presence of critical-like dynamics. For each proximity level ($Z = 0$, 0.5, 1, 1.5, 2.0, 2.5, 3), 400 independent realizations were generated under varying the initial conditions, and a representative Hurst exponent was estimated for the full multivariate system using the average fluctuation function method.

Figure S7 plots the resulting Hurst exponent and error bars. The left panel (B) displays the mean Hurst exponents with standard error across 400 samples, while the right panel (C) shows the corresponding R^2 values, which quantify the quality of the log–log fit. The mean Hurst exponents remain consistently near 1 across all Z. The exponents remain consistently near 1, indicating stable temporal scaling regardless of ES proximity. The uniformly high R^2 values confirm the robustness and reliability of these estimates.

To investigate how phase-coupling influences long-range temporal correlations in complex oscillator systems, we simulated a 78-node Stuart–Landau network under varying levels of PCF. Specifically, we evaluated four conditions—PCF = 25%, 50%, 75%, and 100%—representing increasing levels of phase-synchronized interaction among oscillators. For each condition, we generated 100 independent realizations and computed the Hurst exponent *H* and the corresponding R^2 value of the DFA fit.

As shown in Figure S8, the average Hurst exponent for each PCF level, with standard error bars across 100 samples. *H* increases monotonically with PCF. Reaching ~1 at PCF = 100%, which corresponds to the critical coupling state and reflects super-diffusive behavior with strong long-range temporal correlation. At lower PCF levels, *H* values fall in the 0.5–0.7 range, closer to the memoryless regime of a random walk. The right panel displays the corresponding R^2 values for the DFA fits, which remain consistently high (~0.95) across all PCF levels, confirming the robust power-law scaling of the fluctuation functions regardless of coupling strength.

S4.3. Human EEG: Conscious State Before Anesthesia

FSS of the conscious state before anesthesia: Because EEG recordings contain only a limited number of channels, finite-size scaling could not be applied, as the method requires large system sizes to assess scaling and extract critical exponents reliably.

DCC of the conscious state before anesthesia: To further support the hypothesis that the brain operates near a critical state during the conscious (pre-anesthesia) condition, we extracted size and duration statistics from EEG-derived global activity for a representative subject. Events were identified using a threshold of 2.5σ above baseline, and the corresponding probability distributions were constructed for event size and duration.

Figure S9 displays the power-law fits for an individual. The size distribution followed a power-law with exponent τ = 3.61 (Figure S9 A), while the duration distribution yielded an exponent of α = 3.89 (Figure S9 B). Furthermore, the size–duration relationship conformed to a scaling relation of the form $s \sim d^{\gamma}$, with γ = 1.10 (Figure S9 C), consistent with prior observations in near-critical neural systems.

The Deviation from Criticality Coefficient (DCC) for this subject was measured as approximately 0.20, a value within the criticality-consistent regime observed in our dataset. The convergence of size, duration, and scaling exponents, alongside a low DCC, suggests that this subject's brain dynamics during the pre-anesthesia condition reside near a critical point. These findings are in agreement with the theoretical expectations of critical branching processes and prior empirical reports of avalanche dynamics in cortical activity. Power-law exponents τ (for size) and α (for duration) were estimated using maximum likelihood fitting. Goodness-of-fit was evaluated using the Kolmogorov–Smirnov (KS) statistic and corresponding *p*-values, implemented via the NCC toolbox.

Our findings indicate that the DCC was generally lower in the pre-anesthesia state (mean DCC = 0.22) compared to the anesthesia state (mean DCC = 0.30), suggesting a reduced distance from criticality during the normal conscious state. The estimated size exponents τ ranged from approximately 2.8 to 4.8 before anesthesia, with corresponding KS values mostly below 0.08 and *p*-values around 0.5, indicating strong consistency with power-law

scaling. Duration exponents α were similarly within critical regimes, and the size-duration relationship yielded γ values close to theoretical expectations ($\gamma \approx 1.051$–1.2).

In contrast, anesthesia data showed a subtle but notable shift. The τ and α values tended to decrease, and although p-values remained relatively high (typically $p \geq 0.5$), the higher DCC and smaller exponents suggest a departure from the near-critical regime. In particular, several anesthesia segments exhibited $\tau < 3.0$, and some α values dropped below 3.0, potentially indicating subcritical dynamics. Table S7 and S8 summarizes all measured quantities τ, α, KS statistics, p-values, γ, and scale bounds (x_{min}, x_{max}) for both size and duration distributions.

The results of DFA- Hurst exponent of the conscious state before anesthesia: To assess the presence of long-range temporal correlations (LRTC) in brain activity prior to anesthetic induction, we computed the Hurst exponent (H) using detrended fluctuation analysis (DFA) on EEG signals collected from 16 subjects (Figure S9). As shown in Figure S10, the majority of subjects exhibited H values well above the 0.5 threshold, which indicates uncorrelated noise. Notably, 13 out of 16 subjects showed $H > 0.7$, with several values approaching or exceeding 0.9. Subjects 7 and 13 demonstrated particularly high Hurst exponents of $H = 1.028$ and $H = 1.033$, respectively, suggesting dynamics dominated by persistent memory and self-similarity. These findings support the hypothesis that the awake brain operates near a critical regime, where correlation lengths and memory times diverge.

Alongside Hurst exponent estimation, we computed the R^2 values from the linear fits on the log-log scale in the DFA procedure to assess the reliability of the scaling behavior. As shown in the right panel of Figure S10, all R^2 values exceeded 0.93, with a mean value close to 0.97. This high goodness-of-fit confirms that the EEG fluctuations follow robust power-law behavior across temporal scales.

S4.4. Stock Data: Pre-Crisis Period.

The results of FSS: We analyze daily equity returns over a fixed sample from July 1, 2015, to December 31, 2016, for three markets (the United States, Canada, and Korea) whose sizes are sufficient for FSS analysis. Let $Z \in R^{T\times N}$ denote standardized returns (demeaned, unit variance). Series with excessive missing data are removed; short gaps are linearly imputed. Stock-return correlation matrices embed a pervasive market factor that inflates pairwise dependencies across firms. This global component appears as the leading eigenmode of the correlation matrix and can bias percolation toward trivially synchronized, mean-field–like behavior**.** To focus on sectoral and firm-specific structure, we apply a one-factor singular value decomposition (SVD) deflation to the financial data before network construction, thereby suppressing the market-wide mode (i.e., removing the top principal component). Given the standardized return matrix Z, we compute the sample correlation $\mathrm{C} = \frac{1}{T-1} Z^T Z$ and its spectral decomposition

$$\mathrm{C} = Q\Lambda Q^T, \qquad \Lambda = \mathrm{diag}(\lambda_1,\cdots,\lambda_N), \quad \lambda_1 \geq \cdots \geq \lambda_N.$$

Let q_1 be the leading eigenvector (market mode). Project out q_1 at the return level:

$$f_t = Z_t q_1 \qquad \widetilde{Z_t} = Z_t - f_t q_1^T \;\; (t = 1,\cdots T)$$

then re-standardize each column of $\widetilde{Z_t}$ to unit variance. (Equivalently, deflate C as $\tilde{C}$ =C − $\lambda_1 q_1 q_1{}^T$, reset the diagonal to 1.)

All networks and observables below are computed from the defactorized correlation $\tilde{C}$ unless otherwise noted. To probe finite-size effects, we vary the effective system size by sub-sampling firms without replacement across a market-specific sequence of system sizes N ∈ K (e.g., U.S.: N = {64, 150, 261, 396, 600, 909, 1200}), averaging observables across replicates at each N.

Figure S11 synthesizes the finite-size-scaling readouts for the three markets. In the United States, the cluster-size CCDF at $p_c^*(\mathrm{N})$ exhibits a central slope of ≈ −1.5 (implying τ ≈ 2.5), while size–scaling of the second-largest component and the susceptibility proxy yields $\hat{\omega}_1$ = 0.614 and $\hat{\omega}_2$ = 0.355. This pair is consistent with a *mean-field* percolation signature; the slightly smaller $\hat{\omega}_1$ than the MF reference d_f/d = 2/3 is plausibly explained by density-selection and finite-size effects in correlation networks even after market-mode defactorization. In Canada, the CCDF is flatter in the central regime (slope ≈ −1.0, i.e., τ ≈ 2.0) and the exponents are larger, $\hat{\omega}_1$ = 0.812 and $\hat{\omega}_2$ = 0.748, placing the market closer to a 3*D-like* universality class characterized by higher $\gamma/(\nu d)$ and d_f/d. Korea combines a U.S.-like CCDF slope (≈ −1.5, τ ≈ 2.5) with intermediate size–scaling exponents, $\hat{\omega}_1$ = 0.693 and $\hat{\omega}_2$ = 0.427, which sit between MF and 3D. This pattern suggests an *intermediate* universality tendency, consistent with strong synchronization with the U.S. market (pulling toward MF behavior) alongside residual sectoral/regional clustering that sustains a 3D component. Collectively, the evidence places the United States as MF-like, Canada as 3D-like, and Korea in between; the numerical summaries are reported in Table 9. Taken together, these finite-size-scaling signatures—power-law cluster-size tails at $p_c^*(\mathrm{N})$ and systematic scaling of $\langle N_2 \rangle$and $\langle s \rangle_{\geq 2}$ with N—provide strong evidence that stock markets in the pre-crisis period operate in (or very near) a criticality regime.

Table 9 consolidates the finite-size scaling (FSS) exponents across the Stuart–Landau model, empirical stock markets, and theoretical universality classes. The Stuart–Landau oscillator network recovers the well-known 2D percolation universality class, with (w_1, w_2, τ) ~ (0.99, 0.91, 1.8) closely matching theoretical predictions. This agreement confirms that our FSS procedure reliably identifies criticality when applied to systems with known scaling behavior, providing a methodological benchmark. In contrast, the stock markets exhibit distinct deviations: the United States aligns with mean-field scaling, Canada with a 3D-like regime, and Korea occupies an intermediate position between MF and 3D.

The results of DFA: To assess long-range temporal correlations, a key indicator of criticality, in financial markets, we calculated the average Hurst exponent from daily firm-level volatility for 39 countries using second-order detrended fluctuation analysis (DFA). The data span the pre-crisis period from September 1, 2005, to December 31, 2006. As shown In Figure S12, all estimated Hurst exponents exceed 0.6, with several countries (e.g., IND, RUS, CHN) approaching or surpassing 0.66, indicating strong persistence in return volatility dynamics across diverse markets. These values are substantially above the 0.5 threshold for memoryless stochastic processes (e.g., random walks), supporting the presence of significant temporal correlations consistent with critical-state behavior in complex systems. The bottom panel shows the R^2 values from the DFA log–log fits, all

above 0.93 and mostly near 0.95–0.96, confirming robust power-law scaling and reliable Hurst exponent estimates.

Summary of Criticality Analyses: Finite-size scaling (FSS) of the modified Stuart–Landau model at maximal PCF recovered exponents consistent with the 2D percolation universality class, validating the model's operation in a genuine critical regime and establishing FSS as a methodological benchmark. Complementary avalanche-based metrics confirmed this: the Deviation from Criticality Coefficient (DCC) minimized and branching ratio approached 1 at peak PCF, robustly identifying the critical point across network sizes. Detrended fluctuation analysis (DFA) further showed Hurst exponents near 1, indicating persistent long-range correlations. In human EEG, avalanche statistics during the conscious (pre-anesthesia) state yielded power-law size and duration distributions, low DCC values (~0.2), and high Hurst exponents (>0.7 in most subjects), all consistent with near-critical dynamics. Under anesthesia, exponents and DCC values shifted away from criticality, indicating a departure from this regime. In financial markets, FSS applied to pre-crisis equity returns revealed universality deviations: the U.S. aligned with mean-field scaling, Canada with a 3D-like regime, and Korea exhibited intermediate behavior. Across 39 countries, DFA of firm-level volatility showed Hurst exponents consistently above 0.6, with several exceeding 0.66, confirming persistent long-range temporal correlations.

Taken together, across simulation, EEG, and financial data, multiple independent tests (FSS, avalanche statistics, DCC, branching ratio, and DFA) consistently support critical or near-critical dynamics at maximum PCF in our model, during the conscious state, and in the pre-crisis period.

S5. Test Whether the Deviation from Criticality in The Pre-Crisis Can Explain Criticality Loss and Recovery Times.

We investigate whether deviation from criticality is statistically associated with the brain or financial market's ability to resist or recover from external perturbations. Specifically, we analyze the relationship between long-range temporal correlations (LRTC), quantified by the Hurst exponent, and criticality transition times (e.g., induction or recovery) across two empirical systems: human EEG under anesthesia and national stock markets during global financial crisis.

S5.1 Model data.

Hurst exponent and DCC at peak PCF vs Criticality loss/recovery time. We examined if criticality metrics could predict the criticality loss/recovery time in model simulations set far from explosive synchronization (Z=0), thereby testing their predictive power on stability independent of ES proximity. We specifically tested whether the Deviation from Criticality Coefficient (DCC) and the Hurst exponent were correlated with the time required for the network to lose and subsequently recover its critical state after a perturbation. As illustrated in Figure S13, our analysis found no statistically significant correlations. The relationship between the DCC and the time to lose criticality was not significant (Spearman $\rho = -0.20$, $p = 0.09$), nor was its relationship with recovery time (Spearman $\rho = 0.28$, $p = 0.10$). Similarly, the Hurst exponent did not significantly predict the time to criticality loss (Spearman $\rho = -0.11$, $p = 0.07$) or recovery time (Spearman $\rho = 0.07$, $p = 0.38$). These

findings indicate that these specific criticality measures alone are insufficient for predicting the network model's resilience to disturbances.

S5.2. Human Brain EEG.

DCC of Conscious state vs Consciousness loss/recovery time. We examined whether the proximity to criticality, as quantified by the Dynamic Criticality Coefficient (DCC), is predictive of anesthesia induction or recovery times. Specifically, we performed correlation analyses between DCC values and two physiological markers: the time required to induce anesthesia (induction time) and the time needed to return to wakefulness (recovery time). As shown in Figure S14, no significant relationship was observed in either case. For induction time (Fig. S13 A), the Spearman correlation coefficient was ρ = -0.13 (p = 0.64), indicating a weak and non-significant negative association. For recovery time (Fig. b), the correlation was slightly positive (Spearman ρ = 0.17), but again statistically non-significant (p = 0.53). These results suggest that while DCC is a robust marker of criticality in neural dynamics, it does not reliably predict behavioral state transitions, such as induction or emergence from anesthesia.

Hurst exponent of conscious state vs Consciousness loss/recovery time. To examine whether individual variations in LRTC are associated with anesthetic sensitivity, we computed the Spearman correlation between the Hurst exponent and two behavioral outcomes: induction time (the time to loss of consciousness) and recovery time (the time to regain consciousness). As shown in Figure S15, no statistically significant correlations were found. The relationship between H and induction time yielded a Spearman correlation of $\rho = -0.07$ ($p = 0.80$), while the correlation with recovery time was essentially null ($\rho = -0.003$, $p = 0.99$). These results suggest that while the pre-anesthetic EEG reflects scale-free temporal structure consistent with critical dynamics, this metric does not predict how quickly a subject transitions into or emerges from the anesthetized state. This behavioral dissociation implies that although the conscious brain may exhibit critical-state dynamics, transitions across conscious states—such as induction or emergence—may depend on different physiological mechanisms not directly captured by the Hurst exponent alone.

S5.3. Stock Data

Hurst exponent in pre-crisis period vs response/recovery time. To investigate whether the long-range temporal correlation properties in financial systems are related to crisis resilience, we analyzed stock market data from 39 countries during the 2008 global economic crisis. Using firm-level volatility from 2005–2006, we estimated the Hurst exponent via detrended fluctuation analysis (DFA) and compared it to: (1) Response time: duration from crisis onset to peak market deviation. (2) Recovery time: duration from crisis nadir to return to pre-crisis level.

Table S10 reports Spearman correlation coefficients between the average Hurst exponent of firm-level volatility and the response and recovery times associated with the 2008 global financial crisis. To assess robustness, we computed Hurst exponents using firm-level return volatility across 39 countries over three different pre-crisis baseline periods: starting from July 2005, September 2005, and November 2005, all ending on December 31, 2006. The Hurst exponents were estimated using detrended fluctuation analysis (DFA) of order 2, which captures long-range temporal correlations often associated with criticality in complex systems.

Response and recovery times were computed using various values of the α parameter (ranging from 40 to 120 trading days), which determines the window size for defining the temporal extent of market deviation and rebound following crisis-induced shocks.

Across nearly all configurations, the Spearman correlations are weak and statistically insignificant, with p-values far exceeding conventional thresholds. This result holds for both response and recovery times, irrespective of the α parameter or the baseline window used to compute the Hurst exponent. These findings suggest that the presence of long-memory dynamics—as captured by DFA-derived Hurst exponents—does not robustly explain how quickly or slowly markets respond to or recover from extreme external shocks.

To examine whether long-memory characteristics in firm-level volatility are associated with a market's capacity to absorb and rebound from external crises, we analyzed the relationship between Hurst exponents and market reaction times across 39 countries. The Hurst exponent was calculated from return volatility data over the pre-crisis window (September 1, 2005, to December 31, 2006), where values greater than 0.5 indicate persistent long-range dependence.

Crisis response time and recovery time were defined with respect to the global financial crisis of 2008, using a horizon of $\alpha = 100$ trading days to demarcate the onset, peak deviation, and return to baseline. As shown in Figure S15, no statistically significant association was found between Hurst exponents and either response time (Spearman $\rho = -0.05$, $p = 0.73$) or recovery time (Spearman $\rho = -0.04$, $p = 0.77$). These results suggest that while long memory is a fundamental feature of market volatility dynamics, it does not appear to govern the speed of crisis adjustment, at least during this period.

Across both domains (neural and financial), long-range correlation metrics using Hurst exponents do not exhibit statistically robust associations with criticality loss or recovery times. These findings suggest that while criticality-inspired LRTC may characterize baseline system dynamics, they do not alone predict the speed of transition following external perturbation. Further analysis in Sections S1~3 shows that ES proximity (i.e., a measure for how close a system's phase transition type is to ES – a first-order transition) offers more predictive power for transition timing.

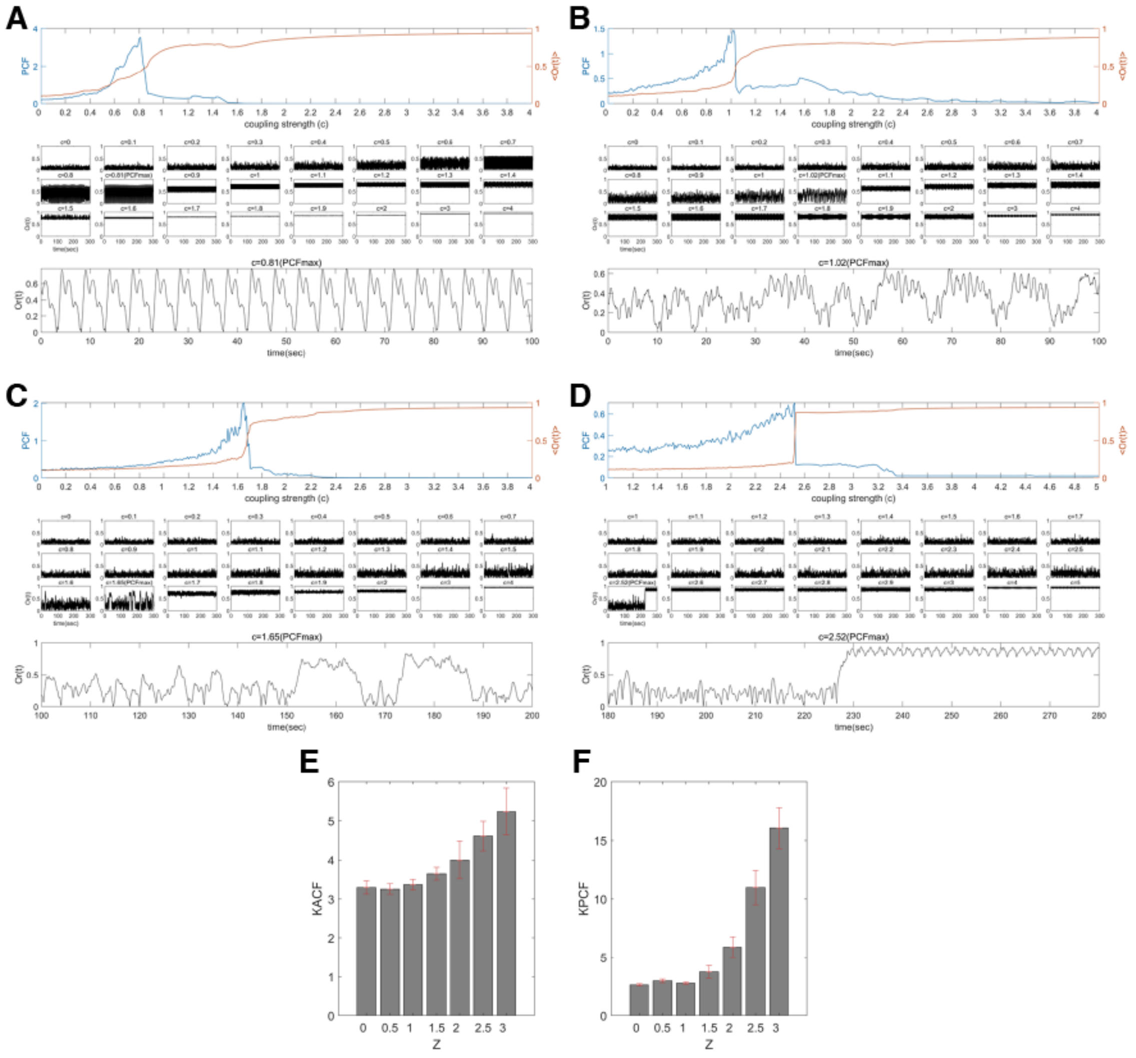

Figure S1. KACF and KPCF of instantaneous order parameters at critical points versus ES proximity (Z) in computational models. Panels (A-D) provide examples illustrating changes in the PCF and the order parameters as coupling strength varies from 0 to 4, displaying characteristic network dynamics for ‘distant’ and ‘close’ ES proximities with adaptive feedback strengths (Z = 0, 1, 2, and 3). The first column shows how PCF changes with different coupling strengths and identifies the maximum PCF as the critical point for each network. The second column shows changes in the order parameter over time (300 seconds) for different coupling strengths. The third column presents fluctuations in the instantaneous order parameter near the critical point and quantifies its characteristic dynamics using KACF and KPCF. Panels (E) and (F) illustrate changes in KACF and KPCF as functions of ES proximity (Z) in the model, with error bars indicating standard errors.

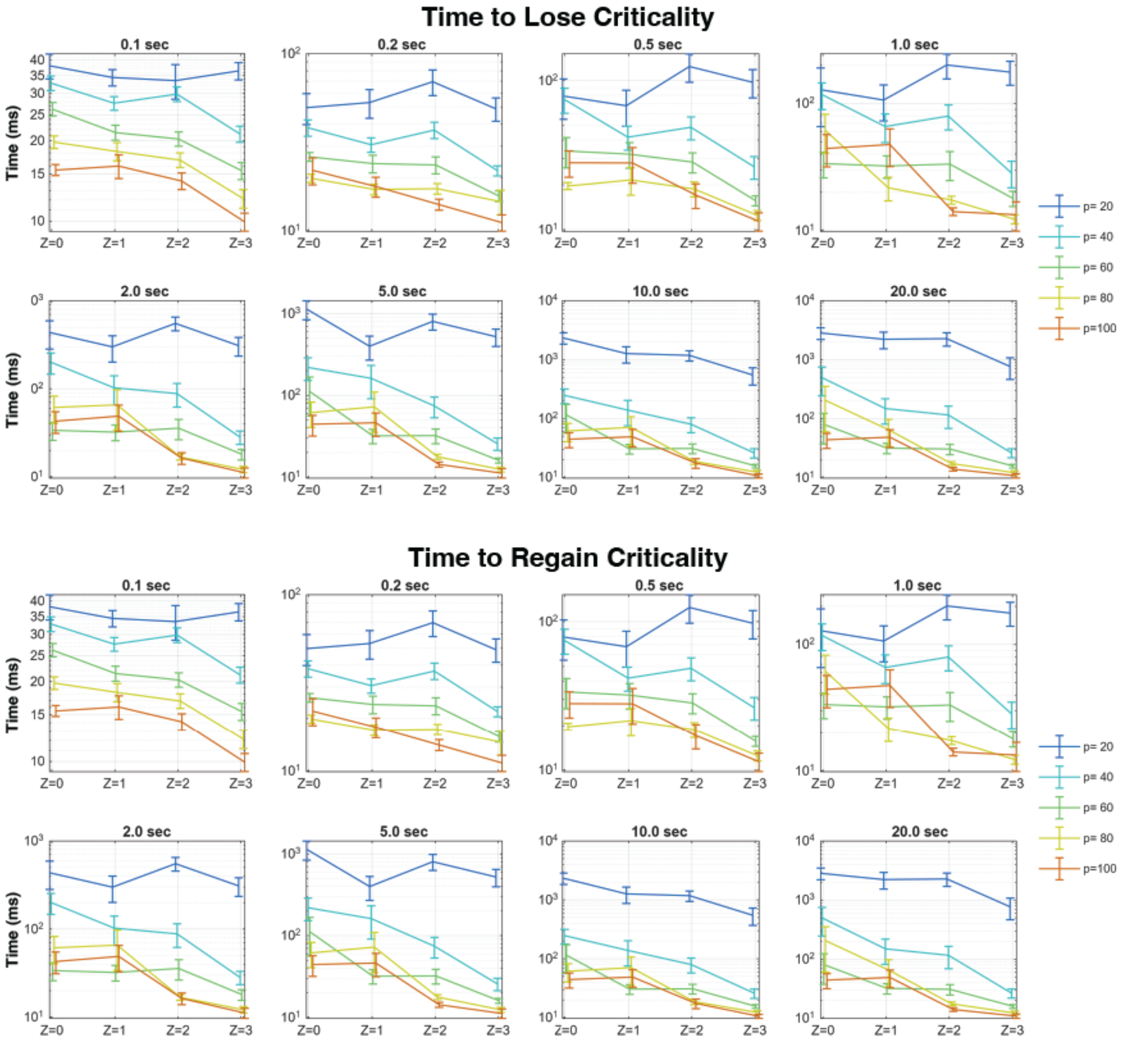

Figure S2. **Relationships between ES proximity (adaptive feedback strength, Z), stimulation strength, stimulation duration, and the times to critical state loss and recovery.** Networks with closer ES proximity (larger Z) exhibit shorter times to critical state loss and longer times to critical state recovery. This pattern is consistent across stimulation strengths (*u*=20, 40,60,80,100) and stimulation durations (0.1, 0.2, 0.5,1, 2, 5, 10, 20 seconds), except in cases where the stimulation is too weak (*u*=20) to deviate from the baseline dynamics. The relationship between ES proximity (*Z*) and the times to critical state loss and recovery becomes salient under external stimulations strong enough to push the network dynamics away from the critical state (u>20). Notably, the time scales of critical state loss and recovery depend on both the stimulation strength and duration.

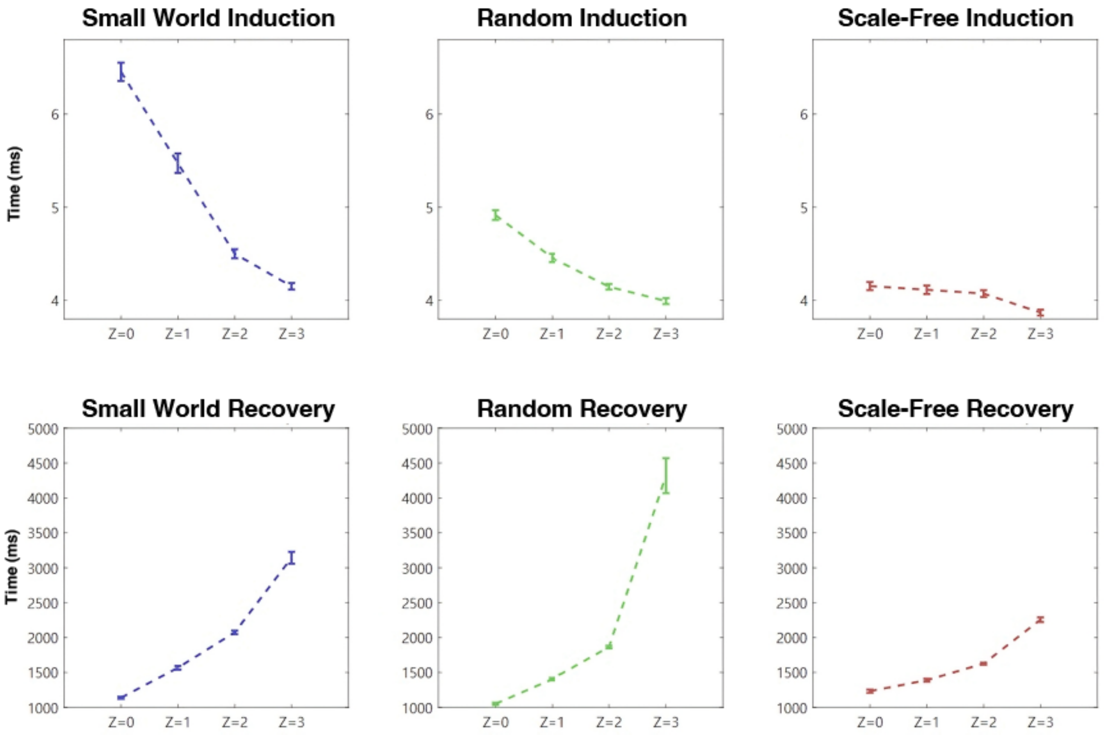

Figure S3. **Relationships between ES proximity (adaptive feedback strength, Z), network topology (small-world, random, scale-free), and times to critical state loss and recovery.**

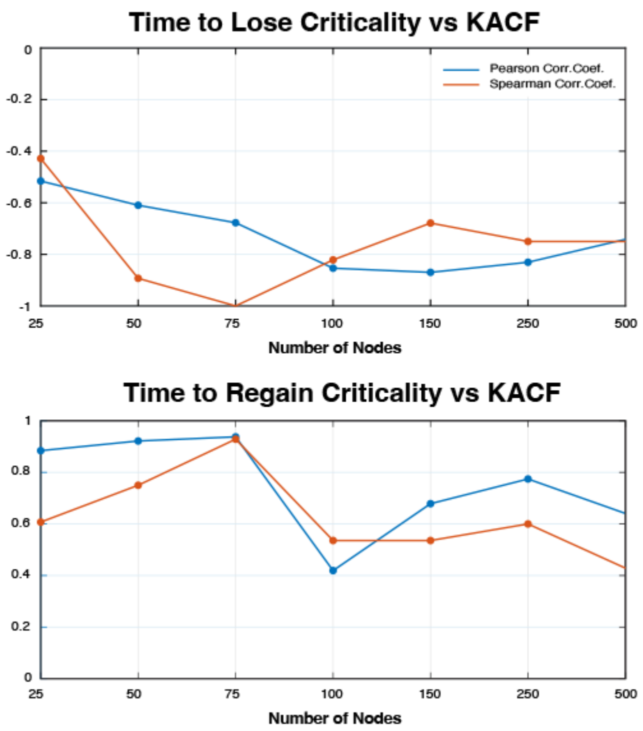

Figure S4. Robustness of correlations against varying network size. The figure displays Pearson (blue) and Spearman (orange) correlation coefficients between KACF and the time to lose criticality (top) and regain criticality (bottom) across random networks of varying sizes (N = 25, 50, 75, 100, 150, 250, and 500 nodes). The stability of the strong correlations across these different scales demonstrates that the correlations are robust to network size.

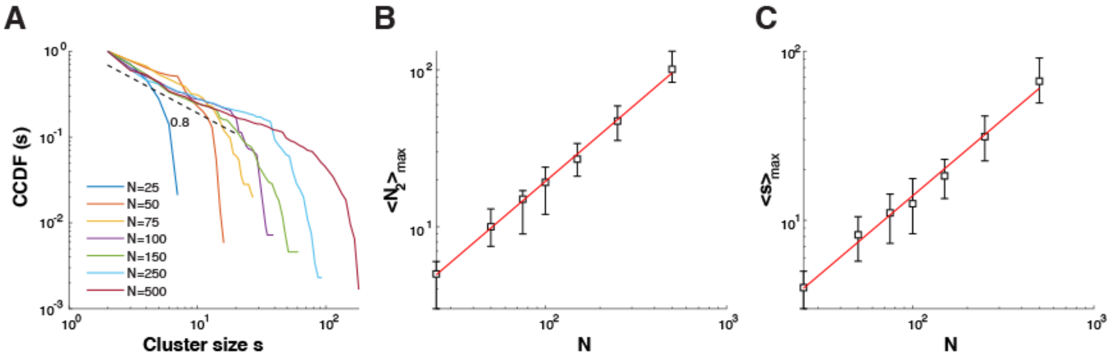

Figure S5. Finite-size scaling at the PLI network of the Stuart–Landau model (at the PCF maximum). (A) Complementary cumulative distribution function (CCDF) of cluster sizes for different system sizes (N). Each colored line represents the pooled CCDF from 100 realizations for a given N at its critical point. The dashed black line indicates a reference power-law with an exponent of -0.8, corresponding to a Fisher exponent $\tau \approx 1.8$. (B) Scaling of the maximum second-largest cluster size ($< N^2 >_{max}$) with system size N. Data points represent the median across 100 realizations, and error bars indicate the interquartile range. The red line shows the power-law fit, yielding an exponent $\omega_1 \approx 0.990$. (C) Scaling of the susceptibility proxy ($< s >_{max}$) with system size N. Data points and error bars are medians and interquartile ranges, respectively. The red power-law fit yields an exponent $\omega_2 \approx 0.909$. The close agreement between the estimated exponents (ω_1, ω_2, τ) and the theoretical values for the 2D percolation universality class provides evidence for criticality in the model.

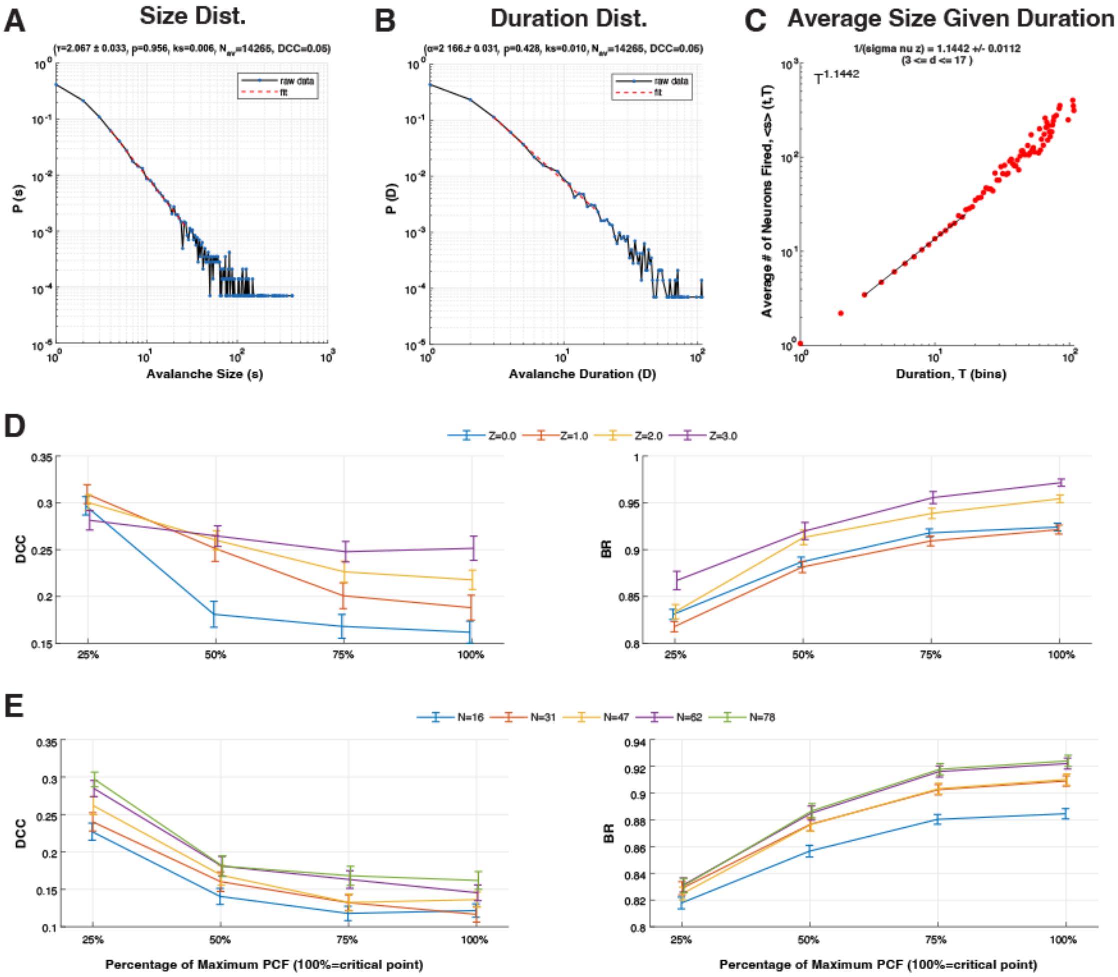

Figure S6. Validation of the PCF-defined critical point using avalanche metrics. (A-C) Representative power-law scaling of avalanches at the 100% PCF state (Z=0). (D) The Deviation from Criticality Coefficient (DCC) is minimized and the Branching Ratio (BR) approaches 1 at the 100% PCF state, a finding robust across different feedback strengths (Z). (E) This scaling relationship is scale-invariant, as the same critical-point characteristics are observed across different sizes of subsampled networks, confirming a key signature of criticality.

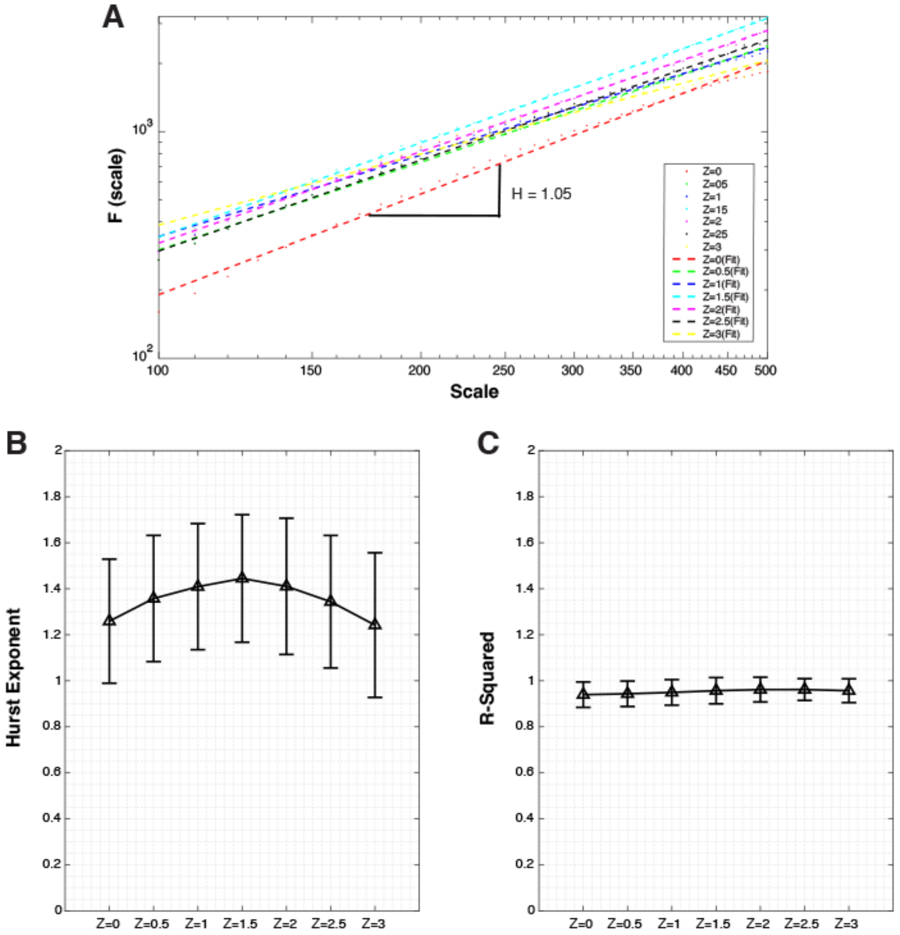

Figure S7. DFA of multivariate time series from a 78-node Stuart-Landau network across ES proximity levels. Multivariate time series generated by a 78-node Stuart–Landau oscillator network were analyzed using second-order DFA. Each colored curve represents the fluctuation function $F(s)$ for a given ES proximity level $Z = 0$ to $Z = 3$. Dashed lines indicate linear fits in log–log coordinates over the scale range $s \in [100, 500]$ (A). All regimes show consistent power-law scaling with Hurst exponents close to 1. Notably, $Z = 0$ yields $H \approx 1.05$, suggesting a long-range persistence characteristic of critical dynamics. The average Hurst exponent (B) and corresponding R^2 *values* (C) *across* 400 random realizations for each Z. The average Hurst exponent remains consistently near one across ES proximity levels, and all fits achieve high R^2, confirming the statistical reliability of the DFA estimates.

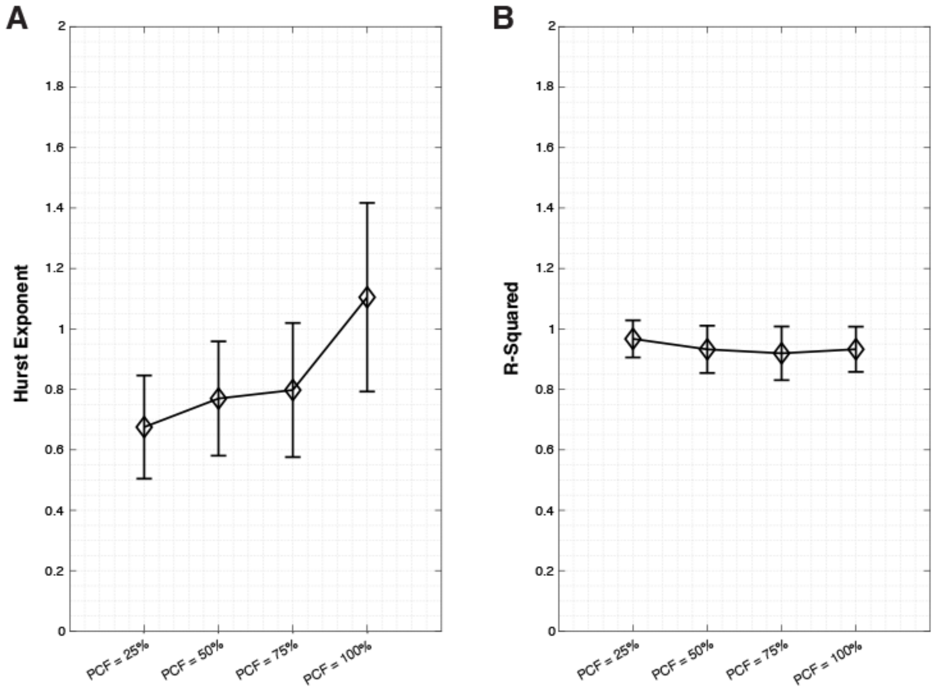

Figure S8. PCF modulates temporal memory in the Stuart–Landau network. The left panel displays the mean Hurst exponent across 100 trials for each PCF condition (25%, 50%, 75%, and 100%). As PCF increases, the Hurst exponent increases, indicating stronger long-range correlations. At PCF = 100%, the system reaches a critical state with $H > 1.0$, consistent with super-diffusive behavior. The right panel shows the R^2 values of the DFA fits. The high R^2 across all conditions confirms the validity of power-law scaling. These results suggest that stronger phase coupling enhances temporal memory, making a transition toward critical dynamics.

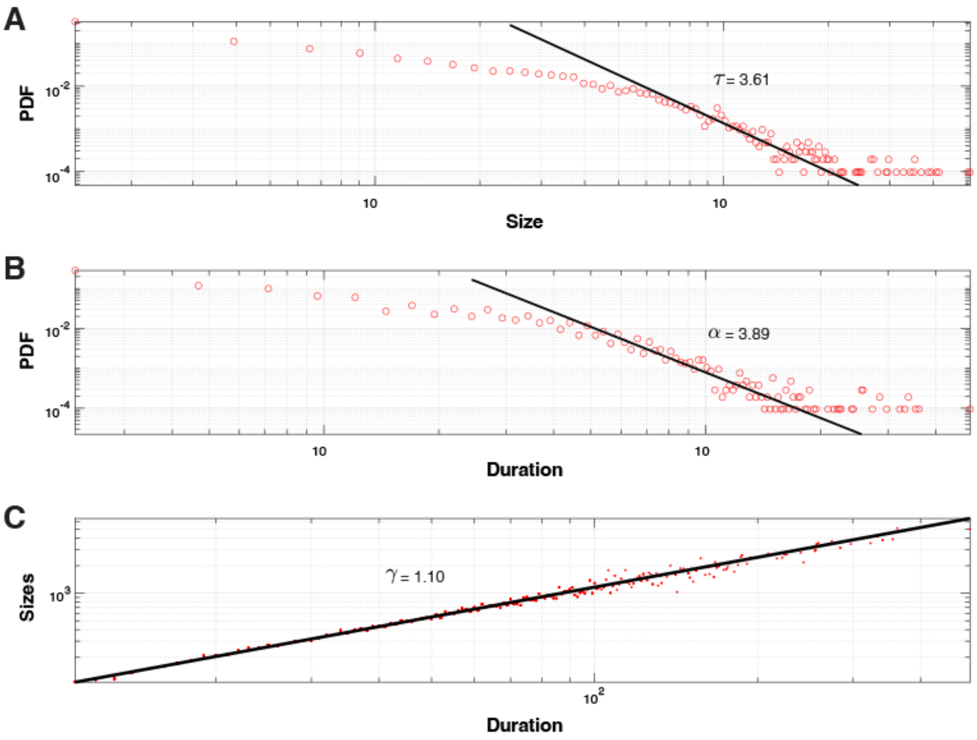

Figure S9. Power-law scaling in a single subject during the pre-anesthesia state. (A) Size distribution with exponent τ = 3.61. (B) Duration distribution with exponent α = 3.89. (C) Size–duration scaling with exponent γ = 1.10. All exponents were fitted using maximum likelihood estimation on the thresholded EEG event data. To quantify criticality in neural dynamics, we analyzed EEG time series data under two conditions: pre-anesthesia (baseline) and post-anesthesia. The EEG signals were normalized to have a zero mean and unit variance, and events were defined as threshold crossings exceeding 2.5 standard deviations (σ). Global neural activity was estimated from these events, from which we extracted both event *size* and *duration* distributions. We then computed the Dynamic Criticality Coefficient (DCC) to evaluate proximity to criticality, alongside power-law scaling exponents and associated statistical fits.

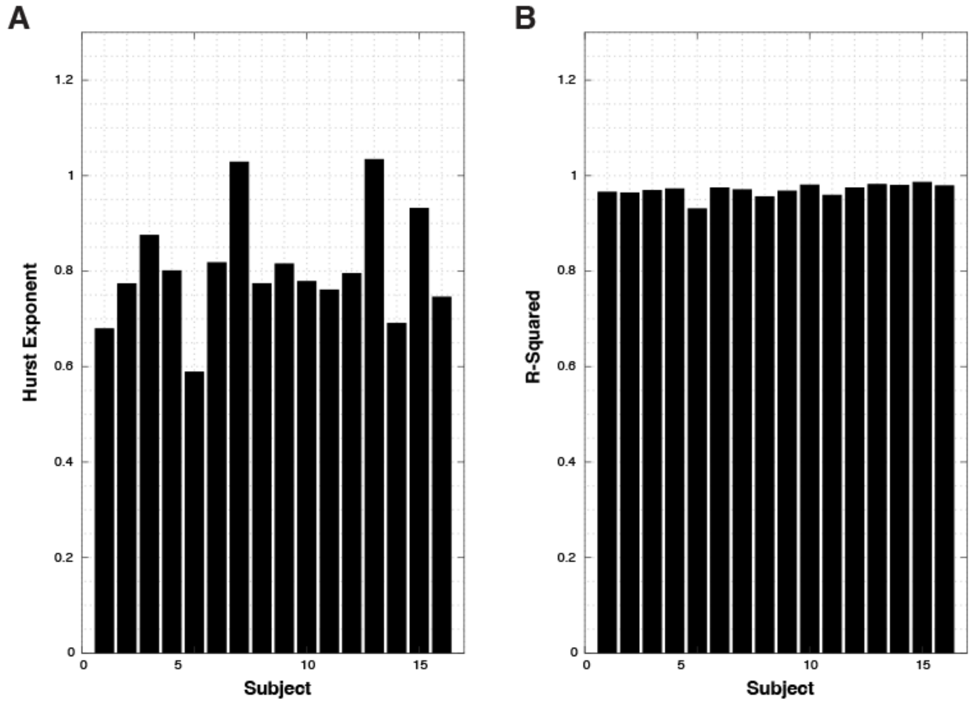

Figure S10. Hurst exponent and R-squared values of pre-anesthetic EEG signals across 16 subjects. The left panel shows the Hurst exponents estimated from the EEG time series recorded prior to anesthetic administration. Most subjects display $H > 0.7$, with several reaching values above 0.9. Notably, subjects 7 and 13 exhibit $H = 1.028$ and $H = 1.033$, respectively, indicating strong long-range temporal correlations (LRTC) consistent with critical-state brain dynamics. The right panel presents the corresponding R^2 values from the log-log linear fits of detrended fluctuation analysis. All values exceed 0.93, with a mean around 0.97, supporting the reliability of power-law scaling in the fluctuation functions. Together, these results indicate that pre-anesthetic brain activity exhibits temporal organization characteristic of criticality, with persistent memory and scale-invariant structure across individuals.

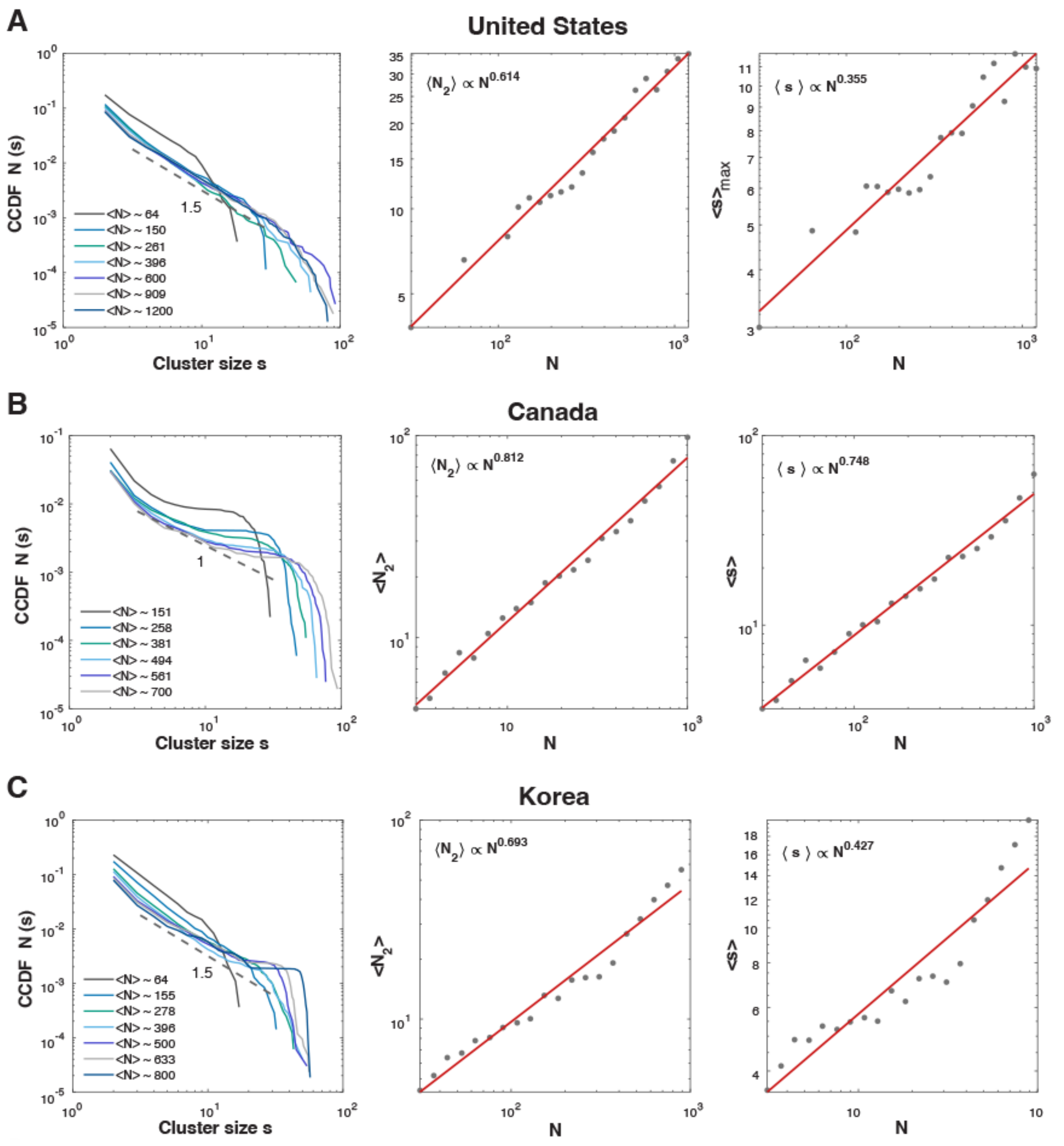

Figure S11. Finite-size scaling (FSS) in equity correlation networks (July 2015 – December 2016). Each row shows diagnostics at the estimated critical threshold $p_c^*(\mathrm{N})$. For the United States (A), the CCDF and scaling slopes (ω_1, ω_2) = (0.614, 0.355) align with mean-field (MF) percolation, reflecting strong global synchronization. For Canada (B), flatter CCDFs and slopes (0.812, 0.748) approach 3D percolation, consistent with geometry-constrained clustering. Korea (C) lies between MF and 3D, with slopes (0.693, 0.427) indicating partial synchronization with the U.S. alongside residual clustering from domestic structural concentration. Together, these results highlight distinct universality regimes across markets and reveal how global linkage versus local segmentation shapes critical dynamics.

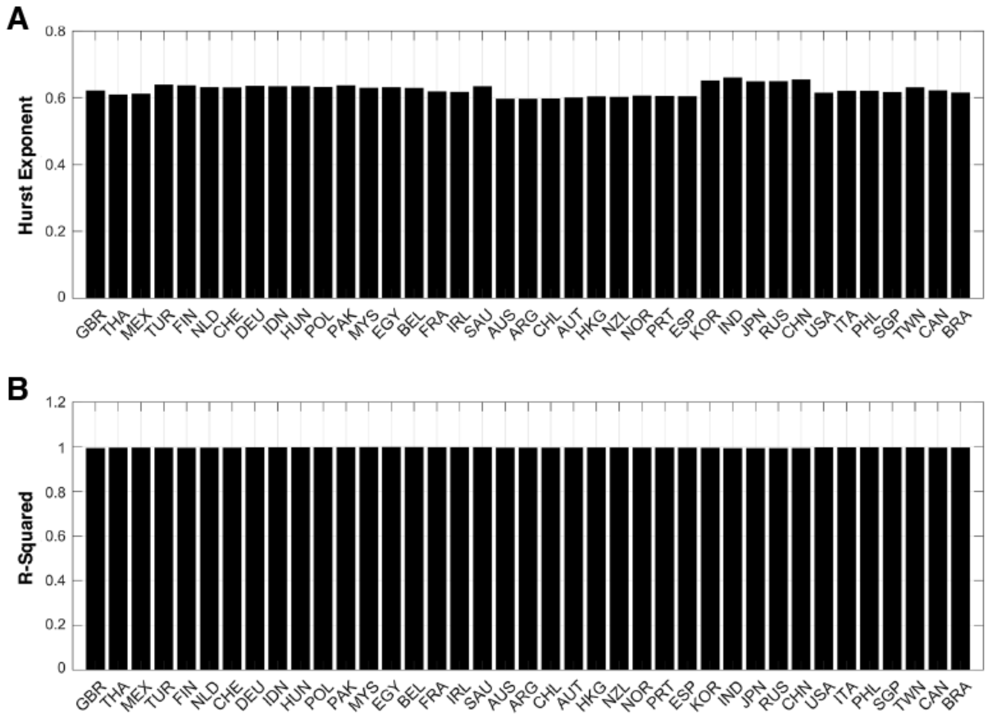

Figure S12. Cross-country comparison of Hurst exponent and goodness-of-fit for firm-level volatility. The top panel displays the average Hurst exponent for daily return volatility across 39 countries from September 2005 to December 2006. All values exceed 0.6, indicating strong long-range memory in market fluctuations. The bottom panel shows the corresponding R^2 values from the DFA log–log scaling fits. All exceed 0.93, with most clustering near 0.95–0.96, validating the reliability of the Hurst exponent estimates. These results suggest that persistent volatility dynamics are a near-universal feature of modern financial systems and may reflect proximity to criticality.

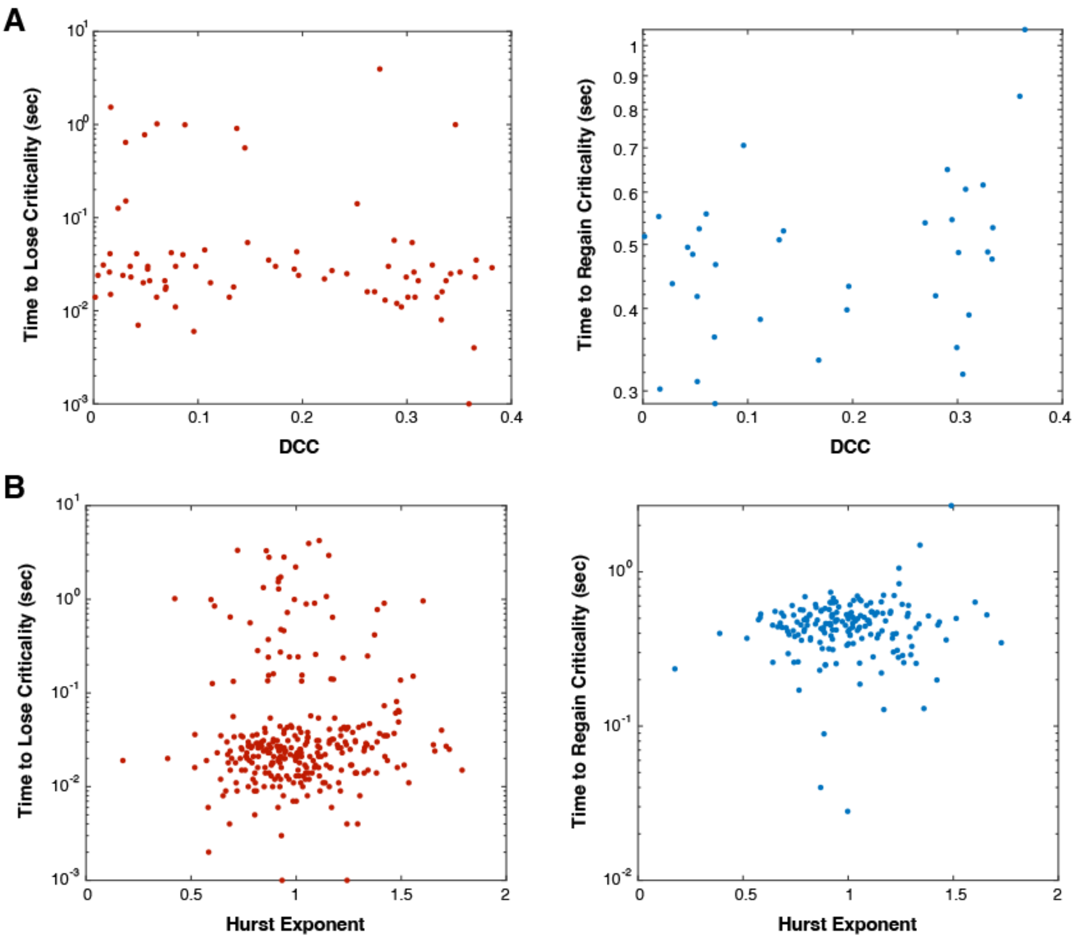

Figure S13. Correlations of DCC and Hurst exponent with time to criticality loss and recovery (Z=0). (A) DCC from 100 iterations against criticality loss and recovery times (Left: Spearman $\rho = -0.20$, $p = 0.09$, Right: Spearman $\rho = 0.28$, $p = 0.10$). (B) Hurst exponent from 400 iterations against the same stability measures (Left: Spearman $\rho = -0.11$, $p = 0.07$, Right: Spearman $\rho = 0.07$, $p = 0.38$). These results suggest that metrics of deviation from criticality are insufficient to predict the times to criticality loss and recovery under external perturbations.

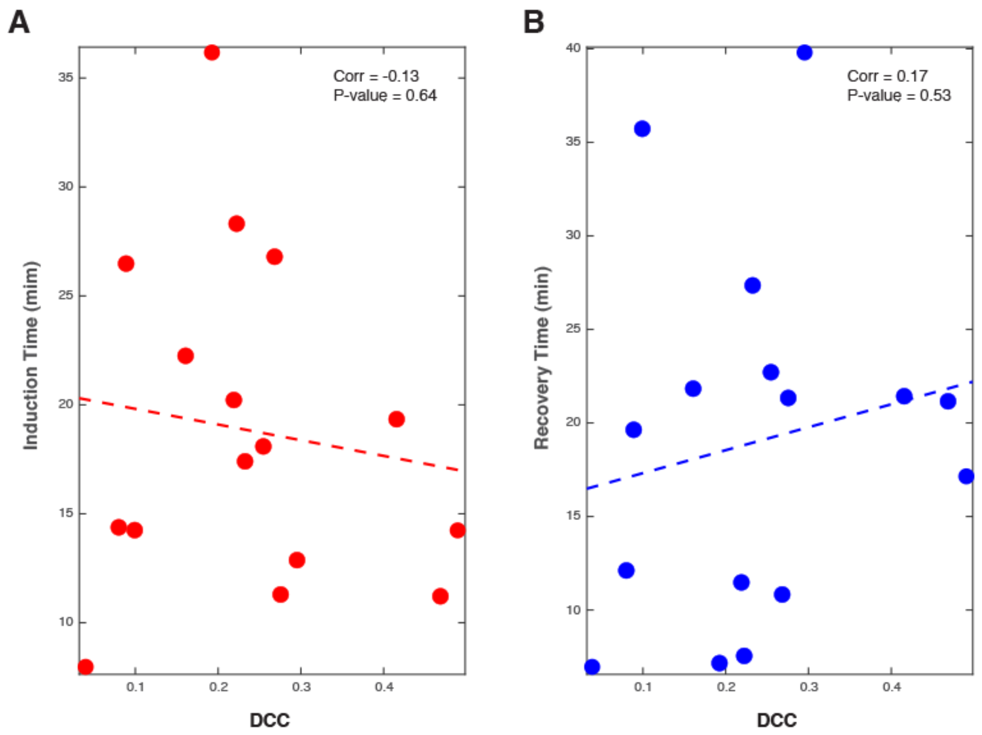

Figure S14. Correlation between DCC and consciousness loss and recovery times in anesthesia. (A) Induction time versus DCC (Spearman $\rho = -0.13$, $p = 0.64$). (B) Recovery time versus DCC (Spearman $\rho = 0.17$, $p = 0.53$). DCC does not show a statistically significant correlation with either transition metric.

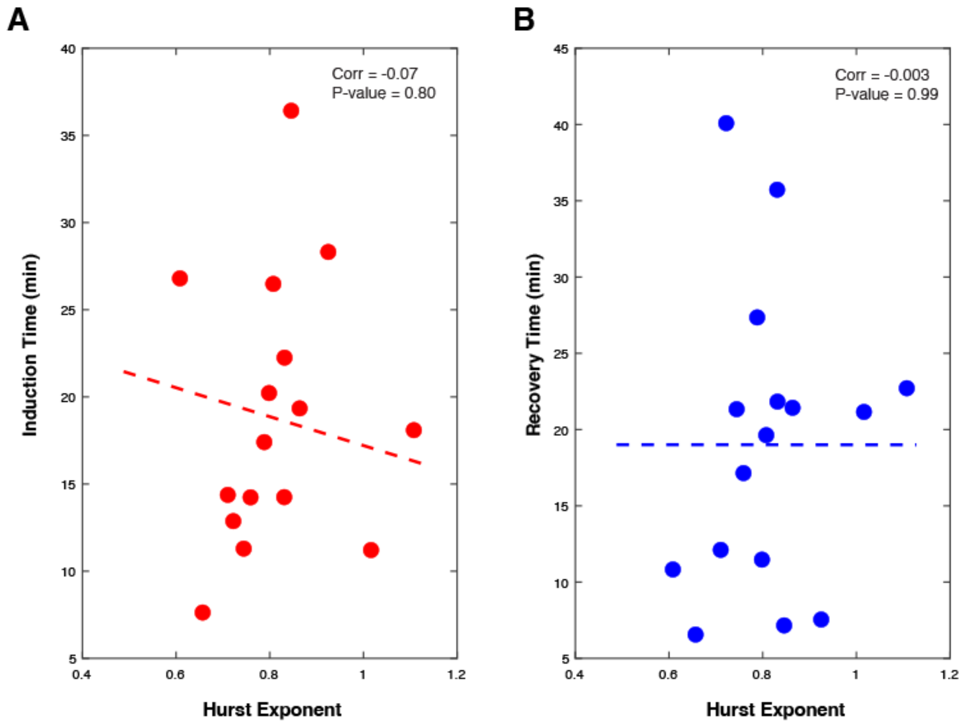

Figure S15. Correlation between Hurst exponent and consciousness loss and recovery times in anesthesia. The left panel (A) shows the correlation between Hurst exponent and induction time (Spearman $\rho = -0.07$, $p = 0.80$). The right panel (B) depicts the correlation between Hurst exponent and recovery time ($\rho = -0.003$, $p = 0.99$). No significant correlations were observed in either case, suggesting that pre-anesthetic long-range temporal correlations cannot account for consciousness loss and recovery times in anesthesia.

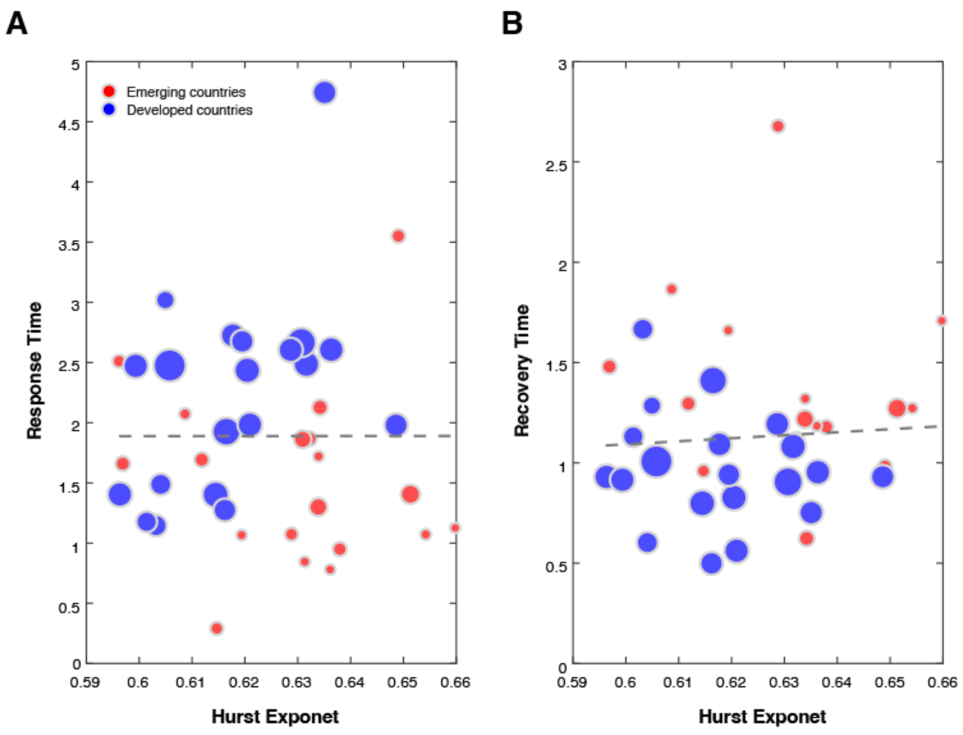

Figure S16. Spearman correlation between Hurst exponents and market response/recovery times during economic crisis. Scatter plots illustrating the relationship between the average Hurst exponent of firm-level volatility and market reaction times during the 2008 global financial crisis. Hurst exponents were computed from firm-level return volatility data for 39 countries over the period from September 1, 2005, to December 31, 2006. The panel (A) shows the correlation between Hurst exponents and response time—the time required for markets to reach peak deviation after the onset of crisis. The panel (B) shows the correlation with recovery time—the time required for markets to return to pre-crisis levels. Both measures were computed using a time window defined by α = 100 trading days. Spearman correlation analysis reveals no statistically significant relationship: response time shows a correlation of ρ = -0.05 (p = 0.73), and recovery time shows ρ = 0.05 (p = 0.77). These findings suggest that the degree of long memory in pre-crisis volatility does not systematically predict how rapidly a market responds to or recovers from strong economic shocks.

Table S1. Figure S1 (E), one-way ANOVA and post-hoc analysis, Multi-comparisons – 'LSD' and 'Tukey-Kramer' (Significant differences between groups are highlighted in gray): F(2,793) = 26.46.

KACF: Multi-comparison – 'LSD'

Z	Z	difference	Lower 95%	Upper 95%	p-value
0	0.5	-0.644	-0.143	0.358	0.577
0	1	-0.780	-0.280	0.221	0.275
0	1.5	-0.963	-0.463	0.0380	0.0705
0	2	-1.107	-0.606	-0.105	0.0178
0	2.5	-1.917	-1.416	-0.915	3.25e-08
0	3	-3.119	-2.619	-2.118	3.14e-24
0.5	1	-0.638	-0.137	0.364	0.593
0.5	1.5	-0.821	-0.320	0.181	0.211
0.5	2	-0.964	-0.464	0.0370	0.069
0.5	2.5	-1.774	-1.274	-0.773	6.58e-07
0.5	3	-2.977	-2.476	-1.975	7.16e-22
1	1.5	-0.684	-0.184	0.317	0.473
1	2	-0.828	-0.327	0.174	0.201
1	2.5	-1.638	-1.137	-0.636	8.91e-06
1	3	-2.84	-2.339	-1.839	9.96e-20
1.5	2	-0.645	-0.144	0.357	0.574
1,5	2.5	-1.455	-0.954	-0.453	0.000193
1.5	3	-2.657	-2.156	-1.656	4.98e-17
2	2.5	-1.311	-0.810	-0.310	0.00153
2	3	-2.514	-2.013	-1.512	4.69e-15
2.5	3	-1.704	-1.203	-0.702	2.63e-06

KACF: Multi-comparison - 'Tukey-Kramer'

Z	Z	difference	Lower 95%	Upper 95%	p-value
0	0.5	-0.896	-0.143	0.610	0.998
0	1	-1.033	-0.280	0.473	0.930
0	1.5	-1.216	-0.463	0.290	0.541
0	2	-1.359	-0.606	0.147	0.211
0	2.5	-2.169	-1.416	-0.663	6.15e-07
0	3	-3.372	-2.619	-1.865	0
0.5	1	-0.890	-0.137	0.616	0.998
0.5	1.5	-1.073	-0.320	0.433	0.874
0.5	2	-1.217	-0.464	0.289	0.539
0.5	2.5	-2.027	-1.274	-0.521	1.28e-05
0.5	3	-3.229	-2.476	-1.723	2.36e-22
1	1.5	-0.937	-0.184	0.569	0.992
1	2	-1.08	-0.327	0.426	0.862
1	2.5	-1.89	-1.137	-0.384	0.000174
1	3	-3.092	-2.339	-1.586	1.19e-19
1.5	2	-0.897	-0.144	0.609	0.998
1,5	2.5	-1.707	-0.954	-0.201	0.00357
1.5	3	-2.909	-2.156	-1.403	2.05e-16
2	2.5	-1.563	-0.810	-0.0570	0.0255
2	3	-2.766	-2.013	-1.26	3.63e-14
2.5	3	-1.956	-1.203	-0.450	5.14e-05

Table S2. Figure S1 (F), one-way ANOVA and post hoc analysis, F(2,793) = 149.89

KPCF: Multi-comparison – ‘LSD'

Z	Z	difference	Lower 95%	Upper 95%	p-value
0	0.5	-1.280	0.07700	1.433	0.9110
0	1	-1.329	0.02800	1.385	0.9670
0	1.5	-2.101	-0.7440	0.6120	0.2820
0	*2*	-5.109	-3.752	-2.395	6.420e-08
0	*2.5*	-9.930	-8.573	-7.216	2.380e-34
0	*3*	-16.975	-15.618	-14.261	9.830e-104
0.5	1	-1.406	-0.04900	1.308	0.9440
0.5	1.5	-2.178	-0.8220	0.5350	0.2360
0.5	*2*	*-5.186*	*-3.829*	*-2.472*	*3.450e-08*
0.5	*2.5*	-10.007	-8.650	-7.293	6.350e-35
0.5	*3*	-17.052	-15.695	-14.338	1.160e-104
1	1.5	-2.130	-0.7730	0.5840	0.2640
1	*2*	*-5.138*	*-3.781*	*-2.424*	*5.100e-08*
1	*2.5*	-9.959	-8.602	-7.245	1.450e-34
1	*3*	-17.003	-15.646	-14.29	4.440e-104
1.5	*2*	*-4.365*	*-3.008*	*-1.651*	*1.430e-05*
1,5	*2.5*	-9.186	-7.829	-6.472	4.800e-29
1.5	*3*	-16.231	-14.874	-13.517	5.920e-95
2	*2.5*	-6.179	-4.822	-3.465	4.030e-12
2	*3*	-13.223	-11.866	-10.509	9.690e-63
2.5	*3*	-8.402	-7.045	-5.688	6.350e-24

KPCF: Multi-comparison - 'Tukey-Kramer'

Z	Z	difference	Lower 95%	Upper 95%	p-value
0	0.5	-1.964	0.07700	2.117	1
0	1	-2.012	0.02800	2.069	1
0	1.5	-2.785	-0.7440	1.296	0.9360
0	2	-5.792	-3.752	-1.712	1.230e-06
0	2.5	-10.613	-8.573	-6.533	0
0	3	-17.658	-15.618	-13.577	0
0.5	1	-2.089	-0.04900	1.991	1
0.5	1.5	-2.862	-0.8220	1.219	0.9000
0.5	2	-5.869	-3.829	-1.789	6.530e-07
0.5	2.5	-10.691	-8.650	-6.610	0
0.5	3	-17.735	-15.695	-13.655	0
1	1.5	-2.813	-0.7730	1.267	0.9230
1	2	-5.821	-3.781	-1.741	9.710e-07
1	2.5	-10.642	-8.602	-6.562	0
1	3	-17.687	-15.646	-13.606	0
1.5	2	-5.048	-3.008	-0.9680	0.0002790
1,5	2.5	-9.870	-7.829	-5.789	0
1.5	3	-16.914	-14.874	-12.834	0
2	2.5	-6.862	-4.822	-2.781	5.650e-11
2	3	-13.906	-11.866	-9.826	0
2.5	3	-9.085	-7.045	-5.005	0

Table S3. Details of economic crises in 39 countries.

ISO	COUNTRY	Market Index	#Stocks	Start(S)	End(E)	Max	Min	Emerging market	GDP
GBR	UK	FTSE 100	886	20080101	20090631	20070615	20090303		44590
THA	THAILAND	SET Index	231	20071101	20090531	20071029	20081029	O	3370
MEX	MEXICO	S&P_BMV IPC	46	20080601	20090531	20071018	20081027	O	9457
TUR	TURKEY	BIST 100	276	20080101	20090331	20071015	20081120	O	7961
FIN	FINLAND	OMX Helsinki 25	93	20080201	20090631	20070713	20090309		41309
NLD	NETHERLANDS	AEX	129	20080501	20090601	20070716	20090309		44936
CHE	SWITZERLAND	SMI	165	20080501	20090531	20070601	20090309		59221
DEU	GERMANY	DAX	347	20080301	20090631	20070716	20090306		36894
IDN	INDONESIA	IDX Composite	70	20080501	20090531	20080109	20081028	O	1766
HUN	HUNGARY	Budapest SE	16	20080601	20090631	20070723	20090312	O	11483
POL	POLAND	WIG20	147	20080301	20090731	20071029	20090217	O	9032
PAK	PAKISTAN	Karachi 100	42	20071101	20090531	20080418	20090126	O	1032
MYS	MALAYSIA	KLCI	493	20071101	20090531	20080111	20081028	O	6351
EGY	EGYPT	EGX 30	53	20071101	20090531	20080505	20090205	O	1564
BEL	BELGIUM	BEL 20	95	20080301	20090431	20070523	20090306		38842
FRA	FRANCE	CAC 40	444	20080201	20090631	20070601	20090309		37796
IRL	IRELAND	ISEQ Overall	23	20071201	20090831	20070601	20090309		53739
SAU	SAUDI ARABIA	Tadawul All Share	51	20071101	20090531	20080112	20090309	O	17827
AUS	AUSTRALIA	S&P_ASX 200	573	20080201	20110231	20071101	20090306		40675
ARG	ARGENTINA	S&P Merval	24	20071101	20090531	20071031	20081121	O	5976
CHL	CHILE	S&P CLX IPSA	36	20080301	20090701	20070703	20081010	O	9410
AUT	AUSTRIA	ATX	45	20080301	20090731	20070709	20090309		40675
HKG	HONG KONG	Hang Seng	129	20071101	20090531	20071030	20081027		28028
NZL	NEWZEALAND	NZX 50	47	20071101	20090531	20071002	20090303		26220
NOR	NORWAY	Oslo OBX	90	20071101	20100831	20070719	20081121		74256
PRT	PORTUGAL	PSI 20	30	20080301	20090431	20070723	20090303		19840
ESP	SPAIN	IBEX 35	114	20080301	20090831	20071108	20090309		28414

KOR	KOREA	KOSPI	936	20080201	20090531	20071031	20081024	O	21731
IND	INDIA	Nifty 50	729	20081001	20090331	20080108	20081027	O	802
JPN	JAPAN	Nikkei 225	2664	20080301	20090431	20070709	20090310		36022
RUS	RUSSIA	MOEX Russia	12	20071101	20090531	20071212	20081024	O	7416
CHN	CHINA	Shanghai Composite	162	20080101	20090231	20071016	20081104	O	2095
USA	USA	S&P 500	1723	20071101	20090531	20071009	20090309		46217
ITA	ITALY	FTSE MIB	229	20080301	20090531	20070518	20090309		33448
PHL	PHILIPPINES	PSEI Composite	47	20071101	20090531	20071008	20081028	O	1471
SGP	SINGAPORE	STI	217	20071101	20090531	20071011	20090310		33768
TWN	TAIWAN	TWII	820	20071101	20090531	20071030	20081120	O	16893
CAN	CANADA	S&P_TSX	1070	20070901	20090731	20070719	20090309		40559
BRA	BRAZIL	Bovespa	75	20080601	20090331	20080520	20081027	O	6067

This table provides detailed information regarding the stock market and recession periods for 39 countries analyzed in this study. The first and second columns contain the ISO country codes and full names of the countries, respectively. The third and fourth columns specify the benchmark stock index used for analysis in each country and the number of companies analyzed from each country's stock market. The fifth and sixth columns indicate the start and end dates of the recession periods for each country, as per the OECD recession indicator. For the countries without the recession indicator, the U.S. recession period is used as a reference. The seventh and eighth columns provide the dates when the stock markets of each country reached their maximum and minimum values during the analysis period, capturing the peaks and troughs in market performance. The last two columns represent the emerging market indicator and the GDP per capita (US dollar) for each country from the International Monetary Fund in 2006

Table S4. The robustness of varying baseline periods and α

		alpha(α)								
		40	50	60	70	80	90	100	110	120
Response time	200511	-0.08	-0.12	-0.23	-0.36**	-0.40**	-0.43***	-0.38**	-0.34**	-0.34**
(p-value)		(0.63)	(0.47)	(0.15)	(0.03)	(0.01)	(0.01)	(0.02)	(0.03)	(0.03)
	200509	-0.11	-0.16	-0.27	-0.38**	-0.43***	-0.45***	-0.40**	-0.37**	-0.37**
		(0.49)	(0.33)	(0.10)	(0.02)	(0.01)	(0.00)	(0.01)	(0.02)	(0.02)
	200507	-0.10	-0.14	-0.25	-0.37**	-0.41***	-0.44***	-0.39**	-0.35**	-0.35**
		(0.56)	(0.40)	(0.13)	(0.02)	(0.01)	(0.01)	(0.02)	(0.03)	(0.03)
Recovery time	200511	0.42***	0.36**	0.41**	0.31*	0.37**	0.51***	0.47***	0.40**	0.40**
(p-value)		(0.01)	(0.02)	(0.01)	(0.06)	(0.02)	(0.00)	(0.00)	(0.01)	(0.01)
	200509	0.43***	0.35**	0.40**	0.28*	0.36**	0.50***	0.45***	0.37**	0.37**
		(0.01)	(0.03)	(0.01)	(0.09)	(0.03)	(0.00)	(0.00)	(0.02)	(0.02)
	200507	0.42***	0.36**	0.41**	0.30*	0.37**	0.51***	0.47***	0.39**	0.39**
		(0.01)	(0.03)	(0.01)	(0.06)	(0.02)	(0.00)	(0.00)	(0.01)	(0.01)

This table shows the Spearman correlations between response(recovery) time using different alpha (α) and KACF2 estimated from the different normal periods. KACF2 means kurtosis of autocorrelation function with time lag 2. ***, **, and * represent significant levels at 1%, 5%, and 10%, respectively. Values in parentheses are t-statistics. The parameter α indicates the duration after the minimum (maximum) point of the stock market index, which is used to define both the response time and recovery time in Equation (S10). Three different baseline periods were tested.

Table S5. The robustness to varying time periods (α)

Criticality Indicators	alpha(α)								
	40	50	60	70	80	90	100	110	120
Panel A: Response time (p-value)									
KPCF	-0.49***	-0.45***	-0.26	-0.30*	-0.30*	-0.39**	-0.37**	-0.41**	-0.32**
	(0.00)	(0.00)	(0.11)	(0.07)	(0.06)	(0.02)	(0.02)	(0.01)	(0.04)
KACF1	0.15	0.09	-0.04	-0.07	-0.14	-0.06	-0.06	0.02	-0.08
	(0.37)	(0.60)	(0.81)	(0.68)	(0.41)	(0.70)	(0.73)	(0.91)	(0.62)
KACF2	-0.13	-0.15	-0.27	-0.38**	-0.43***	-0.45***	-0.40**	-0.37**	-0.38**
	(0.45)	(0.36)	(0.10)	(0.02)	(0.01)	(0.00)	(0.01)	(0.02)	(0.02)
KACF3	-0.28*	-0.21	-0.28*	-0.45***	-0.53***	-0.53***	-0.59***	-0.54***	-0.50***
	(0.09)	(0.19)	(0.08)	(0.00)	(0.00)	(0.00)	(0.00)	(0.00)	(0.00)
Panel B: Recovery time (p-value)									
KPCF	0.28*	0.19	0.20	-0.03	0.15	0.22	0.26	0.18	0.15
	(0.08)	(0.25)	(0.23)	(0.83)	(0.35)	(0.19)	(0.12)	(0.27)	(0.36)
KACF1	0.07	0.02	0.16	0.03	0.17	0.24	0.10	0.13	0.07
	(0.69)	(0.92)	(0.33)	(0.84)	(0.29)	(0.15)	(0.53)	(0.42)	(0.68)
KACF2	0.44***	0.37**	0.40**	0.28*	0.35**	0.50***	0.49***	0.42***	0.43***
	(0.01)	(0.02)	(0.01)	(0.08)	(0.03)	(0.00)	(0.00)	(0.01)	(0.01)
KACF3	0.26	0.17	0.22	0.01	0.06	0.21	0.23	0.21	0.12
	(0.12)	(0.30)	(0.19)	(0.96)	(0.72)	(0.19)	(0.16)	(0.21)	(0.47)

This table shows the Spearman correlations between response(recovery) time using different alpha (α) and KACF(KPCF). KACF1 means kurtosis of autocorrelation function

with time lag 1. KPCF means kurtosis of the pair correlation function. ***, **, and * represent significant levels at 1%, 5%, and 10%, respectively. Values in parentheses are t-statistics. The parameter α indicates the duration after the minimum (maximum) point of the stock market index, measured at daily frequency, which is used to define both the response time and recovery time in Equation (S10).

Table S6. Spearman correlations between GDP and KACF(KPCF) with adjusting normal period

	Normal period						
ES proximity	200507 ~ 200612	200508 ~ 200612	200509 ~ 200612	200510 ~ 200612	200511 ~ 200612	200512 ~ 200612	200601 ~ 200612
kacf2	-0.19	-0.32**	-0.33**	-0.30*	-0.35**	-0.30*	-0.36**
(p-value)	(0.26)	(0.05)	(0.04)	(0.06)	(0.03)	(0.07)	(0.02)
kpcf	-0.36**	-0.33**	-0.37**	-0.43***	-0.35**	-0.33**	-0.35**
(p-value)	(0.03)	(0.04)	(0.02)	(0.01)	(0.03)	(0.04)	(0.03)

This table shows the Spearman correlations between GDP per capita and KACF2(KPCF). KACF2 means the kurtosis of the autocorrelation function with a time lag of 2. KPCF means kurtosis of the pair correlation function. ***, **, and * represent significant levels at 1%, 5%, and 10%, respectively. Values in parentheses are t-statistics. GDP represents the country's GDP per capita, which is an indicator of a country’s standard of living.

Table S7: Estimated criticality metrics across pre-anesthesia EEG data.

DCC	τ	KS-value	p-value	α	KS-value	p-value	γ	$\tau(x_{min})$	$\alpha(x_{min})$	$\tau(x_{max})$	$\alpha(x_{max})$
0.30	3.24	0.06	0.50	4.00	0.10	0.00	1.04	868.62	75.05	1826.61	155.37
0.23	4.09	0.09	0.02	4.55	0.06	0.50	1.10	1224.09	92.78	2614.70	195.66
0.42	3.17	0.08	0.05	3.80	0.03	0.50	1.19	980.09	91.46	2181.81	199.09
0.16	4.65	0.07	0.50	4.00	0.08	0.50	1.06	1407.45	118.56	3121.06	260.77
0.27	3.40	0.04	0.50	2.77	0.06	0.50	1.09	1213.40	102.02	2575.55	213.78
0.03	5.19	0.11	0.50	4.75	0.08	0.50	1.09	852.57	78.21	1819.60	164.38
0.47	2.82	0.04	0.50	3.89	0.08	0.01	1.10	500.12	50.06	1082.55	105.44
0.50	3.16	0.06	0.50	4.48	0.08	0.12	1.12	953.30	80.34	2073.30	172.03
0.10	2.74	0.05	0.50	2.42	0.08	0.05	1.13	722.94	68.16	1570.34	145.28
0.19	3.61	0.04	0.50	3.86	0.03	0.50	1.11	803.51	69.76	1726.58	147.42
0.28	3.41	0.03	0.50	3.98	0.05	0.50	1.08	879.64	79.55	1856.59	165.07
0.22	3.83	0.06	0.50	4.09	0.07	0.50	1.14	2688.98	205.60	5643.10	428.12
0.25	4.28	0.08	0.01	4.82	0.04	0.50	1.11	788.91	70.76	1704.62	149.77
0.08	4.79	0.13	0.17	4.28	0.11	0.50	1.08	1274.13	111.22	2764.22	239.66
0.22	3.57	0.04	0.50	3.82	0.04	0.50	1.13	1122.41	100.57	2435.22	214.95
0.09	4.64	0.05	0.50	4.04	0.07	0.11	1.11	1116.66	97.11	2415.56	207.72

Table S8: Estimated criticality metrics across anesthesia EEG data.

DCC	τ	KS-value	p-value	α	KS-value	p-value	γ	$\tau(x_{min})$	$\alpha(x_{min})$	$\tau(x_{max})$	$\alpha(x_{max})$
0.149	2.827	0.064	0.022	2.774	0.047	0.500	1.179	2576.140	173.831	5597.056	373.354
0.287	2.974	0.041	0.500	3.133	0.047	0.500	1.212	2678.380	175.533	5789.331	374.950
0.363	2.061	0.027	0.500	2.264	0.030	0.500	1.203	2073.041	146.155	4518.033	312.943
0.073	3.429	0.055	0.500	3.201	0.032	0.500	1.177	2976.028	202.955	6452.825	435.764
0.347	3.028	0.039	0.500	3.554	0.051	0.500	1.141	1667.867	118.813	3495.200	245.773
0.379	2.307	0.037	0.500	2.683	0.037	0.500	1.156	1815.594	127.806	3919.880	271.336
0.387	2.475	0.032	0.500	2.838	0.048	0.078	1.190	1498.314	104.967	3264.706	224.353
0.399	1.999	0.026	0.500	2.245	0.064	0.012	1.202	1800.488	126.386	3956.013	273.235
0.457	1.951	0.023	0.500	2.251	0.023	0.500	1.217	1617.293	115.682	3536.047	247.873
0.109	3.850	0.038	0.500	3.364	0.052	0.500	1.096	1202.588	85.895	2500.644	177.115
0.331	3.157	0.039	0.500	3.567	0.061	0.038	1.172	2189.065	158.739	4780.703	342.296
0.032	4.283	0.060	0.500	3.672	0.033	0.500	1.261	3754.900	245.251	8030.449	520.210
0.707	1.438	0.062	0.016	1.985	0.050	0.098	1.151	1844.238	119.278	4048.055	258.122
0.562	2.068	0.038	0.500	2.671	0.035	0.500	1.201	1723.628	112.603	3754.130	240.443
0.140	3.991	0.079	0.032	3.972	0.054	0.500	1.147	2363.532	166.842	4951.209	346.368
0.078	3.241	0.075	0.020	3.078	0.055	0.356	1.156	2087.517	148.164	4451.835	311.581

Table S9: Finite-size-scaling (FSS) exponents for the Stuart–Landau model and selected stock markets, compared with theoretical exponents from different universality classes.

Market	$\hat{\omega}_1$	$\hat{\omega}_2$	τ	Tendency
Stuart-Landau	0.990	0.909	≈ 1.8	2D-like
United States	0.614	0.355	≈ 2.5	Mean-field (MF)
Canada	0.812	0.748	≈ 2.0	3D-like
Korea	0.693	0.427	≈ 2.5	Intermediate (MF ↔ 3D)
Mean-Field Percolation	2/3≈ 0.667	1/3≈ 0.333	5/2=2.5	MF
3D percolation	0.84	0.67	2.15	3D
2D percolation	91/96≈ 0.948	43/48≈ 0.896	187/91≈ 2.055	2D

For each system, $\omega_1 = d_f/d$ and $\omega_2 = \gamma/(\nu d)$ were estimated at $p_c^*(\mathrm{N})$ using $\langle N_2 \rangle$ and $\langle s \rangle_{\geq 2}$, while τ denotes the CCDF central exponent. The Stuart–Landau model serves as a methodological benchmark, reproducing the theoretical 2D percolation universality class. The empirical markets show distinct signatures, mean-field (United States), 3D-like (Canada), and intermediate (Korea), while theoretical reference values for MF, 2D, and 3D percolation universality classes are listed for direct comparison.

Table S10. The robustness of varying baseline periods and α

		alpha(α)								
		40	50	60	70	80	90	100	110	120
Response time	200511	-0.140	0.061	-0.011	0.006	0.006	-0.021	0.001	-0.080	-0.096
(p-value)		(0.394)	(0.711)	(0.950)	(0.973)	(0.969)	(0.901)	(0.994)	(0.628)	(0.558)
	200509	-0.185	0.084	0.018	-0.054	-0.086	-0.102	-0.056	-0.141	-0.201
		(0.259)	(0.612)	(0.911)	(0.743)	(0.601)	(0.536)	(0.734)	(0.392)	(0.219)
	200507	-0.174	0.079	0.021	-0.050	-0.077	-0.081	-0.040	-0.128	-0.182
		(0.289)	(0.633)	(0.899)	(0.762)	(0.641)	(0.625)	(0.808)	(0.438)	(0.267)
Recovery time	200511	-0.009	-0.051	0.060	0.082	0.186	0.131	0.124	0.119	0.041
(p-value)		(0.959)	(0.758)	(0.718)	(0.620)	(0.257)	(0.425)	(0.450)	(0.469)	(0.805)
	200509	0.085	0.007	0.053	0.017	0.093	0.049	0.047	0.028	-0.004
		(0.605)	(0.966)	(0.750)	(0.919)	(0.572)	(0.766)	(0.777)	(0.864)	(0.980)
	200507	0.104	0.020	0.061	0.018	0.109	0.059	0.045	0.013	-0.013
		(0.529)	(0.906)	(0.710)	(0.911)	(0.509)	(0.719)	(0.783)	(0.939)	(0.940)

This table shows the Spearman correlations between response(recovery) time using diff erent alpha and Hurst exponent estimated from the different normal periods. Hurst exponent is measured by detrended fluctuation analysis with order of 2.

***, **, and * represent significant levels at 1%, 5%, and 10%, respectively. Values in par entheses are t statistics.

Reference

1. Deco G, et al. (2017) Single or multiple frequency generators in on-going brain activity: A mechanistic whole-brain model of empirical MEG data. *Neuroimage* **152**:538–550.

2. Deco G, Kringelbach ML, Jirsa VK, Ritter P (2017) The dynamics of resting fluctuations in the brain: Metastability and its dynamical cortical core. *Sci Rep* **7**(1):3097.

3. Kim M, et al. (2021) Criticality creates a functional platform for network transitions between internal and external processing modes in the human brain. *Front Syst Neurosci* **15**:657809.

4. Kim H, Moon JY, Mashour GA, Lee UC (2018) Mechanisms of hysteresis in human brain networks during transitions of consciousness and unconsciousness: theoretical principles and empirical evidence. *PLoS Comput Biol* **14**(8):e1006374.

5. Gong G, et al. (2009) Mapping anatomical connectivity patterns of human cerebral cortex using in vivo diffusion tensor imaging tractography. *Cereb Cortex* **19**(3):524–536.

6. Zhang X, Hu X, Kurths J, Liu Z (2013) Explosive synchronization in a general complex network. *Phys Rev E* **88**(1):010802.

7. Caminiti R, Ghaziri H, Galuske R, Hof PR, Innocenti GM (2009) Evolution amplified processing with temporally dispersed slow neuronal connectivity in primates. *Proc Natl Acad Sci USA* **106**(46):19551–19556.

8. Scheffer M, et al. (2009) Early-warning signals for critical transitions. *Nature* **461**(7260):53–59.

9. Chialvo DR, Cannas SA, Grigera TS, Martin DA, Plenz D (2020) Controlling a complex system near its critical point via temporal correlations. *Sci Rep* **10**(1):12145.

10. Kim M, Lee U (2019) Alpha oscillation, criticality, and responsiveness in complex brain networks. *Netw Neurosci* **3**(3):825–845.

11. Yoon S, Sindaci MS, Goltsev AV, Mendes JFF (2015) Critical behavior of the relaxation rate, the susceptibility, and a pair correlation function in the Kuramoto model on scale-free networks. *Phys Rev E* **91**(3):032814.

12. Barabási A-L, Albert R (1999) Emergence of scaling in random networks. *Science* **286**(5439):509–512.

13. Erdős P, Rényi A (1959) On random graphs. *Publ Math Inst Hung Acad Sci* **5**:17–61.

14. Watts DJ, Strogatz SH (1998) Collective dynamics of 'small-world' networks. *Nature* **393**(6684):440–442.

15. Mhuircheartaigh RN, Warnaby C, Rogers R, Jbabdi S, Tracey I (2013) Slow-wave activity saturation and thalamocortical isolation during propofol anesthesia in humans. *Sci Transl Med* **5**(208):208ra148.

16. Warnaby CE, Sleigh JW, Hight D, Jbabdi S, Tracey I (2017) Investigation of slow-wave activity saturation during surgical anesthesia reveals a signature of neural inertia in humans. *Anesthesiology* **127**(4):645–657.

17. Zamponi N, Zamponi E, Cannas SA, Chialvo DR (2022) Universal dynamics of mitochondrial networks: a finite-size scaling analysis. *Sci Rep* **12**:17074.

18. Ma Z, Turrigiano GG, Wessel R, Hengen KB (2019) Cortical circuit dynamics are homeostatically tuned to criticality in vivo. *Neuron* **104**(4):655–664.e4.

19. Beggs JM, Plenz D (2003) Neuronal avalanches in neocortical circuits. *J Neurosci* **23**(35):11167–11177.

20. Marshall N, et al. (2016) Analysis of Power Laws, Shape Collapses, and Neural Complexity: New Techniques and MATLAB Support via the NCC Toolbox. *Front Physiol* **7**:250.

21. Peng CK, et al. (1994) Mosaic organization of DNA nucleotides. *Phys Rev E* **49**(2):1685–1689.

22. Linkenkaer-Hansen K, Nikouline VV, Palva JM, Ilmoniemi RJ (2001) Long-range temporal correlations and scaling behavior in human brain oscillations. *J Neurosci* **21**(4):1370–1377.’
